# An enhanced CNN with ResNet50 and LSTM deep learning forecasting model for climate change decision making

**DOI:** 10.1038/s41598-025-97401-9

**Published:** 2025-04-24

**Authors:** Ahmed M. Elshewey, Mona M. Jamjoom, Eman H. Alkhammash

**Affiliations:** 1 Department of Computer Science, Faculty of Computers and Information, Suez University, P.O. BOX: 43221, Suez, Egypt; 2 Department of Computer Sciences, College of Computer and Information Sciences, Princess Nourah Bint Abdulrahman University, P.O. Box 84428, Riyadh, 11671 Saudi Arabia; 3 Department of Computer Science, College of Computers and Information Technology, Taif University, P.O. Box 11099, Taif, 21944 Saudi Arabia

**Keywords:** Climate change, CNN, ResNet50, LSTM, Deep learning, Temperature, Wind power, Climate sciences, Climate change

## Abstract

Climate change poses a significant challenge to wind energy production. It involves long-term, noticeable changes in key climatic factors such as wind power, temperature, wind speed, and wind patterns. Addressing climate change is essential to safeguarding our environment, societies, and economies. In this context, accurately forecasting temperature and wind power becomes crucial for ensuring the stable operation of wind energy systems and for effective power system planning and management. Numerous approaches to wind change forecasting have been proposed including both traditional forecasting models and deep learning models. Traditional forecasting models have limitations since they cannot describe the complex nonlinear relationship in climatic data, resulting in low forecasting accuracy. Deep learning techniques have promising non-linear processing capabilities in weather forecasting. To further advance the integration of deep learning in climate change forecasting, we have developed a hybrid model called CNN-ResNet50-LSTM, comprising a Convolutional Neural Network (CNN), a Deep Convolutional Network (ResNet50), and a Long Short-Term Memory (LSTM) model to predict two climate change factors: temperature and wind power. The experiment was conducted using three publicly available datasets: Wind Turbine Scada (Scada) Dataset, Saudi Arabia Weather history (SA) dataset, and Wind Power Generation Data for 4 locations (WPG) dataset. The forecasting accuracy is evaluated using several evaluation metrics, including the coefficient of determination ($\:{\text{R}}^{2}$), Mean Squared Error (MSE), Mean Absolute Error (MAE), Median Absolute Error (MedAE) and Root Mean Squared Error (RMSE). The proposed CNN-ResNet50-LSTM model was also compared to five regression models: Dummy Regressor (DR), Kernel Ridge Regressor (KRR), Decision Tree Regressor (DTR), Extra Trees Regressor (ETR), and Stochastic Gradient Descent Regressor (SGDR). Findings revealed that CNN-ResNet50-LSTM model achieved the best performance, with $\:{\text{R}}^{2}$ scores of 98.84% for wind power forecasting in the Scada dataset, 99.01% for temperature forecasting in the SA dataset, 98.58% for temperature forecasting and 98.35% for wind power forecasting in the WPG dataset. The CNN-ResNet50-LSTM model demonstrated promising potential in forecasting both temperature and wind power. Additionally, we applied the CNN-ResNet50-LSTM model to predict climate changes up to 2030 using historical data, providing insights that highlight its potential for future forecasting and decision-making.

## Introduction

The fast development of industry nowadays, as well as the excessive consumption of traditional energy sources such as oil and natural gas and its causes of pollution highlight the importance of renewable energy. Renewable energy has gained increasing attention globally and has been anticipated to address the energy and environmental crises. Accurate and exact wind speed estimation has become more and more important as wind power is integrated into the electrical markets. Over the facility’s lifetime, a 1% inaccuracy in the predicted wind speed might result in a loss of $12,000,000^1^. The random and uncontrollable nature of wind speed makes forecasting extremely challenging^2^. Wind turbines are powered by wind energy which is then converted into electrical energy according to the fundamentals of wind power generation^3^. To decrease the need for power grids, minimize wind turbine loss, and increase annual energy production (AEP) accurate wind speed forecasts are therefore essential^3^. Moreover, load balancing scheduling and wind farm regulation are just a few applications that precise wind energy forecasting can help with^4^. Ensuring reliable wind speed forecasting requires the development of robust models capable of long-term wind speed prediction^4^. There are two types of wind speed prediction methods: traditional physical prediction models, which use statistical prediction techniques, and emerging artificial intelligence models^5^. Traditional models predict wind speed using physical parameters such as climate and season^6^. However, due to complexity and large calculations considering enormous parameters, it is difficult to achieve efficient and accurate wind prediction. Numerical Weather Prediction (NWP) uses physical rules to simulate weather conditions and make large-scale forecasts^7^. However, their poor spatial resolution prevents them from offering precise and accurate forecasts at specific local areas. With the development of statistical prediction models the shortcomings of physical prediction models are successfully addressed. The shortcomings of physical prediction models are successfully resolved with the development of statistical prediction models. Statistical prediction models, often referred to as data-driven or “black box” models, do not consider physical parameters such as topography, climate, or seasons^7^. Physical-statistical models, which combine NWP and statistical models, are being investigated as another approach to address the restrictions mentioned above^8,9^. Such models leverage data outputs from NWPs as predictor variables in statistical models, resulting in higher accuracy^5^. Statistical models include the Kalman filter method and the persistence method. The persistence model is effective in predicting wind speed for short-term, but it is not suitable for medium- and long-term wind speed prediction as it gives forecast value for the next moment^8,10,11^. The Kalman filter method uses linear equations to create a wind speed prediction model. Therefore, it is effective for linear processes that follow a gaussian distribution and not suitable for complicated wind speed situations^12–16^. Some models employ a hybrid approach, integrating the best features of both physical and statistical approaches^9^. Wind speed machine learning methods are divided into two categories: deterministic methods, which employ algorithms to produce certain values, such as recurrent neural networks (RNN), long short-term memory (LSTM), support vector machine (SVM), back propagation neural network (BPNN), and fuzzy neural networks^17,18^. The second category uses uncertain models to predict wind speed and quantify uncertainty such as Bayesian simulators^9^. A single algorithmic technique for forecasting wind speed may not provide reliable forecasts. As a result, numerous hybrid methods, including combining methods and ensemble methods (EMs), have been developed to improve wind speed prediction capabilities^5^. Predictive performance has been successfully increased through the application of ensemble learning. It combines multiple models to create a more powerful model and performs better when the adopted model does not provide great precision. Advanced ensemble learning models include neural networks reinforcement learning multi-objective optimization and boosting^6^. Several studies have adopted hybrid models to improve the performance of wind predictions. Zhang, Y. M., and Wang, H^6^. adopts the hybrid EWT Empirical Wavelet Transform LSTM-RELM Regularized Extreme Learning Machine, IEWT Inverse Empirical Wavelet Transform model has been constructed and has stronger predictive performance. For one-sample ahead prediction, they were able to obtain a Mean Absolute Percentage Error (MAPE) of 2.52%, which is a notable improvement over the 8.5% MAPE of a LSTM model^6^.

This study proposed a novel hybrid CNN-ResNet50-LSTM model that is used to forecast climate change with a particular emphasis on temperature and wind power. The contributions of this study can be outlined as follows:

1. A novel deep learning model CNN-ResNet50-LSTM is proposed that integrates the strengths of three models: CNN, ResNet50, and LSTM. CNN excels at efficiently extracting relevant features from datasets and identifying spatial relationships in climate data. ResNet50 introduces skip connections or shortcuts that span multiple layers, effectively addressing the vanishing gradient problem and enabling the training of much deeper networks. LSTM is used to capture long-term dependencies and temporal sequences within data, which is crucial for climate data characterized by time-series properties.
2. Unlike other deep learning models that focus on forecasting a single factor in climate change, the CNN-ResNet50-LSTM model is used to enable forecasting of both temperature and wind power, providing a more comprehensive understanding of climate change behavior.
3. Extensive experiment tests and evaluation comparisons of the proposed model are conducted with five other regression models: DR, ETR, DTR, SGDR, and KRR using several metrics, including$\:{\text{R}}^{2}$, MSE, MAE, MedAE, and RMSE. The experimental finding revealed that the developed model provides competitive performance compared to other models.
4. Three different benchmark datasets are used for evaluation: Scada, SA, and WPG. Scada dataset is used for wind power forecasting, SA dataset is used for temperature forecasting, whereas WPG dataset is used for both temperature forecasting and wind power forecasting. The datasets are normalized using Z-score normalization which scales the data points to enable fair comparison. Datasets were divided into three categories: training (70%), validation (15%), and testing (15%).
5. The proposed CNN-ResNet50-LSTM model can forecast climate changes to 2030 using historical data, highlighting its promise for future prediction tasks.

Most existing studies focus on either CNNs, LSTMs, or ResNet-based models independently, whereas our study introduces a novel hybrid CNN-ResNet50-LSTM model that captures both spatial and temporal dependencies in climate data. In addition, most studies have focused only on forecasting wind speed, neglecting wind power and temperature. This study fills that gap by combining both climate factors, giving a more complete view of climate change forecasting. Unlike existing models that mostly deal with short-term forecasts, this study shows the ability to predict climate trends up to 2030, helping support long-term planning.

## Related work

This section discusses several studies and methodologies that are used for wind prediction. Li et al.^7^ built a hybrid model of Empirical Wavelet Transform (EWT), Long Short-Term Memory (LSTM), Regularized Extreme Learning Machine (RELM), and Inverse Empirical Wavelet Transform (IEWT) which achieved stronger predictive performance compared to other single models used in studies of wind speed prediction. They were able to obtain a MAPE of 0.0252 for one-sample ahead prediction. Zhu et al.^19^ employed a hybrid deep learning model to forecast short-term wind speeds based on empirical wavelet transform recurrent neural networks and error correction. Their model performed better than the Autoregressive Integrated Moving Average (ARIMA) model and other models. Yu et al.^10^ combine an Elman Recurrent Neural Network (ERNN) and Wavelet Packet Decomposition (WPD) for wind speed forecasting. The proposed model achieved better results than other models. Liu et al.^8^ employ a discrete wavelet transform and long short-term memory network to predict wind power. The discrete wavelet transform divides wind power data into sub-signals, and an independent LSTM is used for each sub-signal to predict wind power. Yang et al.^11^ proposed a novel numerical weather prediction (NWP) correction mechanism for wind power prediction. A double clustering method was developed to predict wind power across various scenarios based on separating changing weather conditions. The forecasting model was applied to a wind farm in western Inner Mongolia China and improved the accuracy of wind power predictions. RMSE and MAE are reduced by an average of 0.0593 and 0.0482 respectively using the suggested prediction model. While the multiple clustering approach and NWP correction mechanism can be adapted to specific datasets, they might not be suitable for other wind power forecasting situations or regions^12^. Different machine learning and deep learning algorithms are used to improve the performance of wind prediction results. The bidirectional GRU (BiGRU) model is used for predicting wind power. Yu et al.^13^ developed a framework for wind prediction using different algorithms; Random First RF to screen for wind parameters. The variational modal decomposition (VMD) is then optimized using the whale algorithm (WOA), a BiGRU optimized by an attention mechanism is proposed. The attention mechanism improves BiGRU’s focus on essential information. The suggested model improves accuracy and reduces MAPE by 86.81% compared to BiGRU^13^. Table 1 shows various studies that represent the state-of-the-art prediction models for climate change.

**Table 1:** Summary of literature review studies on climate change prediction using deep learning models.

References	Focus	Method	Finding
Li et al.^7^	Wind speed prediction	EWT-LSTM-RELM-IEWT	MAPE = 0.0252
Yang et al.^11^	Wind power prediction	NWP model	RMSE = 0.0593 MAE = 0.0482
Elshewey et al.^17^	Temperature prediction	WD-SARIMAX model	MSE = 2.80 $\:{\text{R}}^{2}$ = 91%
Shams et al.^18^	Temperature prediction	CBR model	$\:{\text{R}}^{2}$ = 92.4%
Oo et al.^20^	Temperature forecasting	Prophetic forecasting regression model	RMSE = 5.7573
Zhang et al.^21^	Temperature prediction	LightGBM model	Accuracy = 84%
Nitsure et al.^22^	Sea level prediction	ANN and GP models	MAE = 0.106
Alawadi et al.^23^	Temperature prediction	Extra trees model	RMSE = 0.058
Liyew et al.^24^	Rainfall prediction	XGBoost	MAE = 3.58 RMSE = 7.85
Nyasulu et al.^25^	Humidity prediction	Ensemble model	RMSE = 29.988 $\:{\text{R}}^{2}$ = 93.35%
Nyasulu et al.^25^	Minimum temperature prediction	Ensemble model	RMSE = 1.0515 $\:{\text{R}}^{2}$ = 90.18%
Nyasulu et al.^25^	Maximum temperature prediction	Ensemble model	RMSE = 1.6591 $\:{\text{R}}^{2}$ = 82.05%
Nyasulu et al.^25^	Rainfall prediction	Ensemble model	RMSE = 0.4100 $\:{\text{R}}^{2}$ = 77.33%
Tarek et al.^26^	Wind power prediction	SFS-PSO	RMSE = 0.00002

## Methodology

**Fig. 1:**
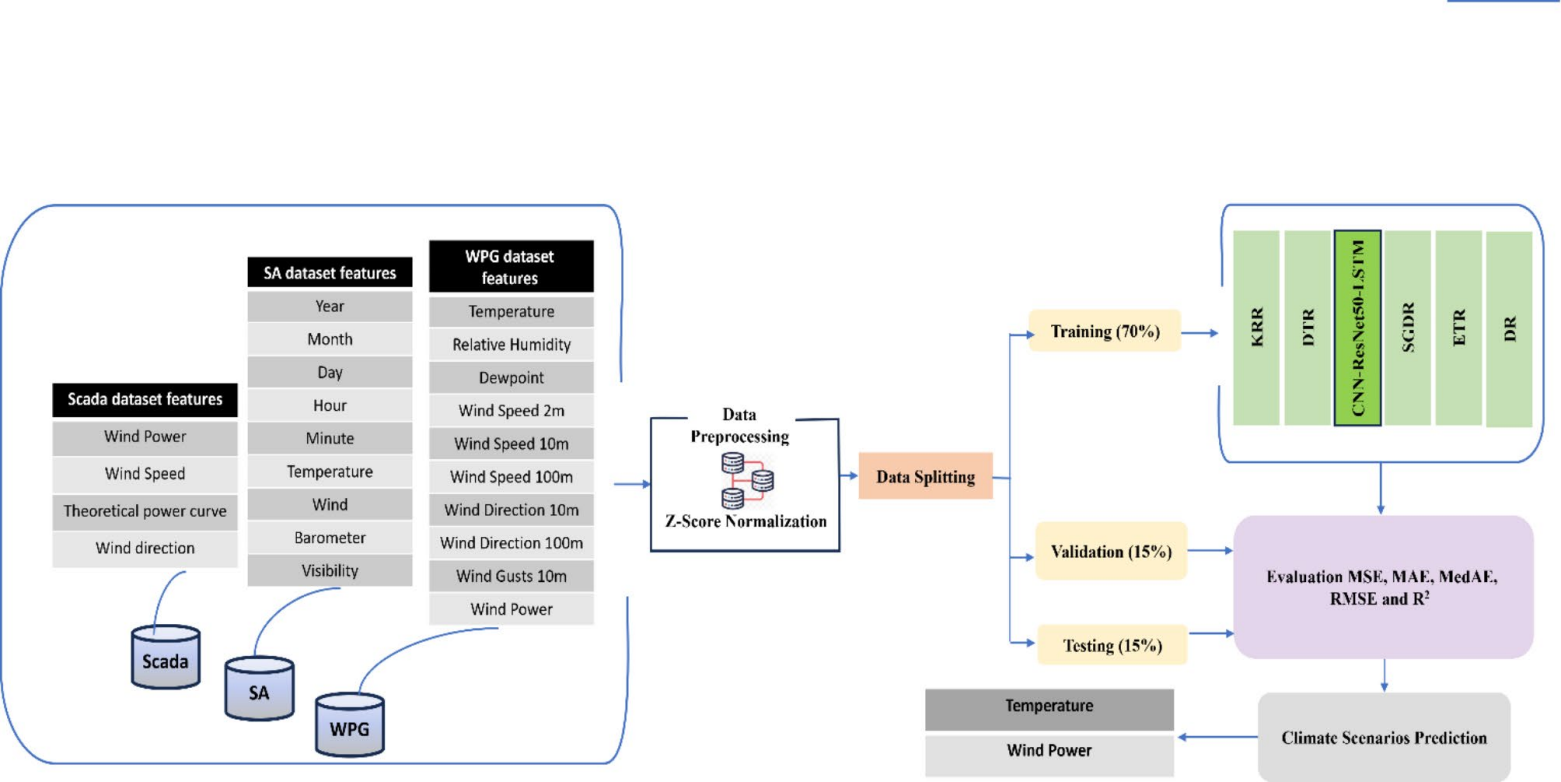
Graphical abstract of the proposed model for temperature and wind power forecast.

The escalating challenges in climate change and their impacts on the environment, economy, and society pose an urgent need for procedures and policies to mitigate its negative impact. The development of deep learning models for effective forecasting of climate change are required to provide an in-depth understanding of climate change behavior. This study enhances our understanding of climate change, with particular emphasis on temperature and wind power, by utilizing deep learning techniques to provide an effective and accurate forecasting model of temperature and wind power. The proposed CNN-ResNet50-LSTM model combines CNN, ResNet50, and LSTM to capture spatial and temporal features, which enables accurate forecasting of temperature and wind power. We evaluate the effectiveness of the CNN-ResNet50-LSTM model by comparing it with five individual regression models: DR, ETR, DTR, SGDR, and KRR. We used several key performance metrics to evaluate the models such as: MSE, MAE, MedAE, RMSE, and R^2^. The experimental results show that the CNN-ResNet50-LSTM model outperforms the five regression models in predicting both temperature and wind power. Figure 1 depicts a graphical abstract of the proposed methodology for forecasting temperature and wind power. The methodology framework consists of the following steps:

1. Dataset features collection.
2. Scaling data using Z-score normalization.
3. Split the dataset into three distinct subsets: 70% for training, 15% for validation, and 15% for testing.
4. Train the CNN-ResNet50-LSTM model and the five regression models.
5. Performance evaluation using several metrics: MSE, MAE, MedAE, RMSE and $\:{\text{R}}^{2}$.
6. Evaluating the model’s performance in forecasting climate change of the two main factors: temperature and wind power.

### Datasets

In this study, we have used three datasets: Scada, SA, and WPG for forecasting temperature and wind power.

#### Wind turbine scada dataset

This dataset is used to predict wind power available at (https://www.kaggle.com/datasets/berkerisen/wind-turbine-scada-dataset).The dataset consists of 50,530 records and 4 features, namely, wind power, wind speed, theoretical power and wind direction. The dataset features are described as follows:

- **Wind_Power (kW)**: “The power generated by the turbine at determined time”.
- **Wind_Speed (m/s)**: “The wind speed used for energy generation”.
- **Theoretical_Power (KWh)**: “The power generated by a turbine at a particular wind speed, as specified by the manufacturer”.
- **Wind Direction (°)**: “The direction of the wind at the turbine’s hub height”.

Table 2 describes the statistical overview of the scada dataset features.

**Table 2:** Statistical overview of the scada dataset features.

Features	Mean	std	Min	50%	Max
Wind_Power	1307.68	1312.4	2.4714	825.83	3618.7
Wind_Speed	7.55795	4.2271	0	7.1045	25.206
Theoretical_Power	1492.17	1368.0	0	1063.77	3600
Wind_Direction	123.687	93.443	0	73.7129	359.99

**Fig. 2:**
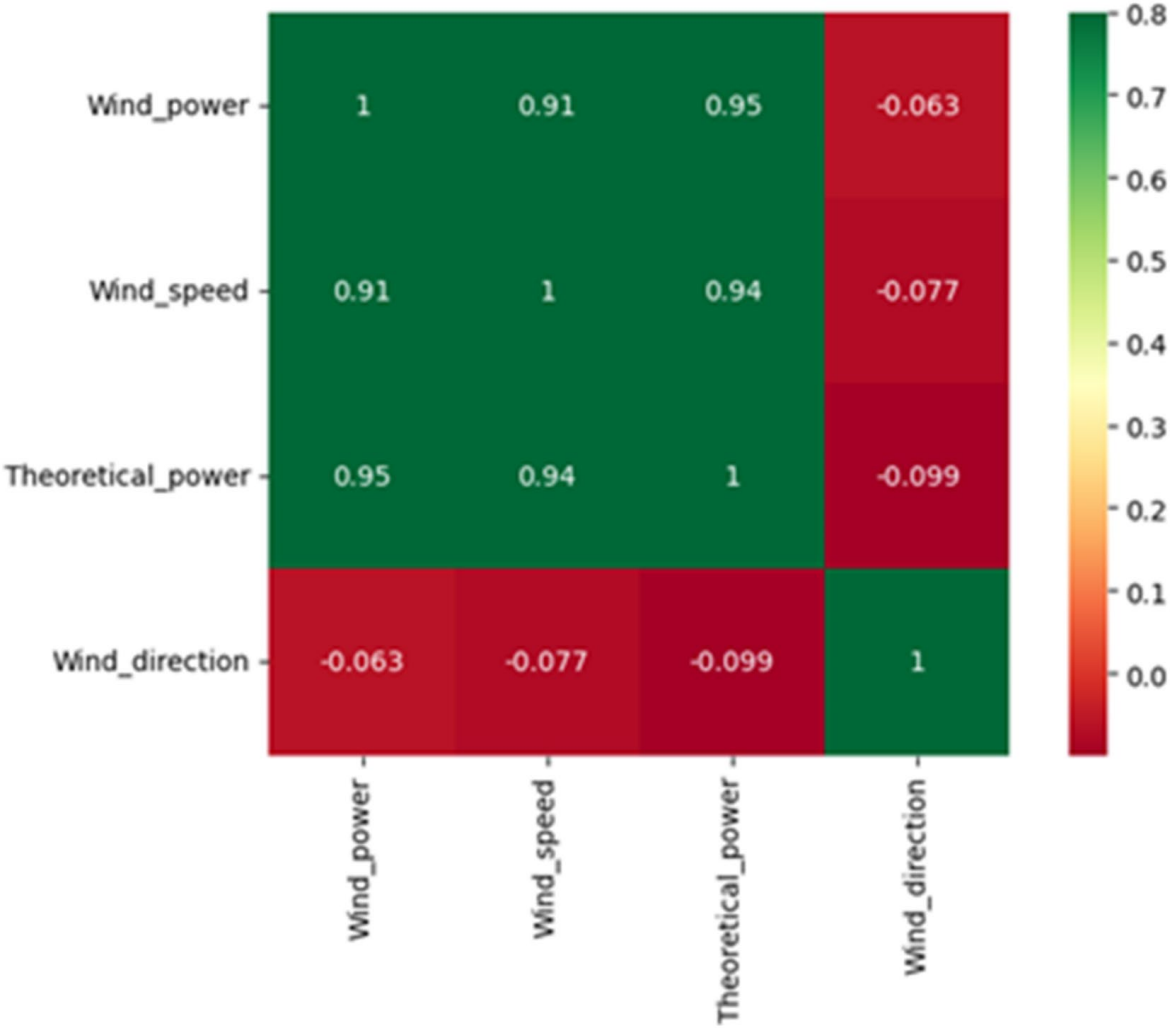
Heatmap analysis of Scada dataset features.

Figure 2 shows the heatmap matrix that represents the correlation between features of the Scada dataset.

#### Saudi Arabia weather history (SA) dataset

This is hourly weather dataset from **2017** to **2019** to predict the temperature available at “https://www.kaggle.com/datasets/esraamadi/saudi-arabia-weather-history”. The dataset consists of 249,023 records and 14 features. The dataset features are described as follows:

- Year: “The year of the data collection”.
- Month: “The month of the data collection”.
- Day: “The day of the data collection”.
- Hour: “The hour of the data collection”.
- Minute: “The minute of the data collection”.
- Temperature: “The average global temperature”.
- Wind speed: “Wind speed determines the velocity of the wind”.
- Visibility: “The distance at which an object or light may be clearly seen”.
- Barometer pressure: “The pressure of air within the atmosphere of Earth”.

Table 3 describes the statistical features of the SA dataset.

**Table 3:** Statistical description of the features of SA dataset.

Features	Mean	Std	Min	50%	Max
Year	2017.7	0.70611	2017.0	2018	2019
Month	6.05069	3.52159	1.0	6	12
Day	15.6910	8.78795	1.0	16	31
Hour	12.5368	6.91025	1.0	13	24
Minute	0.13110	1.97071	0.0	0.0	59
Temperature	24.7226	8.88091	− 4.0	24	50
Wind speed	12.9571	8.71161	− 1.0	11	163
Barometer	1015.45	6.97077	904	1016	1101
Visibility	11.0534	7.05300	− 1.0	16	161

**Fig. 3:**
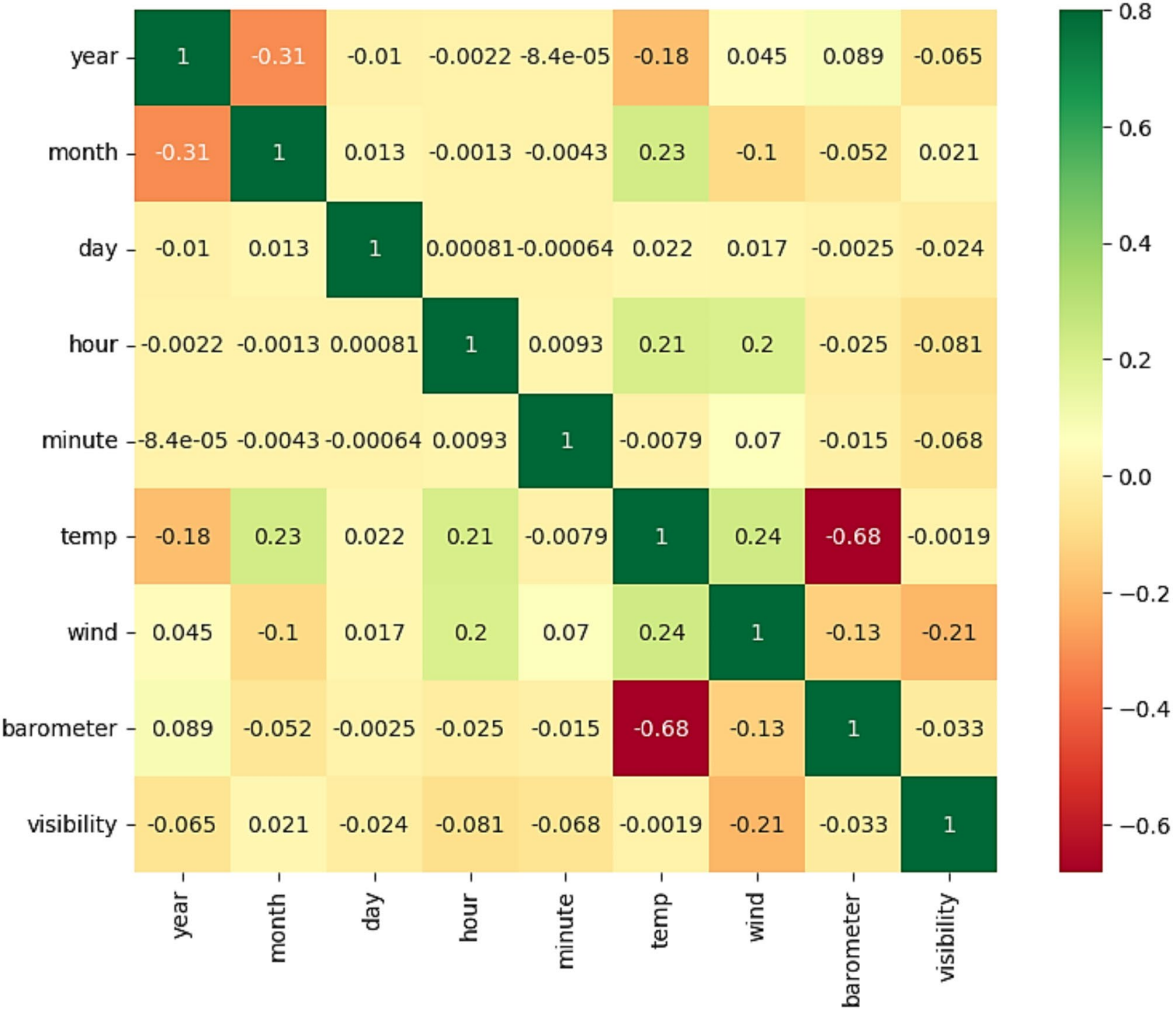
Heatmap analysis of SA dataset features.

Figure 3 shows the heatmap matrix that represents the correlation between features of SA dataset.

#### Wind power generation data for 4 locations (WPG) dataset

The dataset utilized in this study, referenced at^27^, constitutes a time series spanning from 2017 to 2021. It encompasses 9 distinct features and a total of 43,800 records. This extensive dataset provides a robust foundation for developing predictive models aimed at understanding and forecasting climate-related phenomena, particularly focusing on temperature and wind power scenarios. The dataset features are described as follows:

- **Temperature**: “Temperature (degrees Fahrenheit) is measured at a height of 2 m above the ground”.
- **Relativehumidity_2m**: “Relative humidity (percentage) at a height of 2 m”.
- **Dewpoint_2m**: “Dew point in degrees Fahrenheit at a height of 2 m”.
- **Windspeed_10m**: “Wind speed measured at a height of 10 m”.
- **Windspeed_100m**: “Wind speed measured at a height of 100 m above the ground”.
- **Winddirection_10m**: “Wind direction in degrees (0 to 360) at a height of 10 m above the ground”.
- **Winddirection_100m**: “Wind direction in degrees (0 to 360) at a height of 100 m above the ground”.
- **Windgusts_10m**: “Gusts of wind in meters per second at a height of 10 m above the ground”.
- **Wind Power**: “The turbine output has been normalized, scaled so that it is between 0 and 1”.

Table 4 describes the statistical features of the WPG dataset.

**Table 4:** Statistical description of the features of WPG dataset.

Feature name	Count	Mean	Standard deviation	Minimum	Median	Maximum
Temperature	43,800	47.86291	19.45369	− 14.4	47.3000	94.100
Relativehumidity_2m	43,800	72.28874	16.85228	18.0	74.0000	100.00
Dewpoint_2m	43,800	38.56930	18.77211	− 17.1	38.1000	76.300
Windspeed_10m	43,800	3.591147	1.649318	0.0	3.30000	13.450
Windspeed_100m	43,800	6.284431	2.685216	0.1	6.08000	20.650
Winddirection_10m	43,800	203.6373	96.37126	1.0	225.0000	360.00
Winddirection_100m	43,800	203.3436	97.95985	0.0	226.0000	360.00
Windgusts_10m	43,800	7.771795	3.569147	0.5	7.20000	29.200
Wind_Power	43,800	0.405385	0.288322	0.0	0.34765	0.9913

**Fig. 4:**
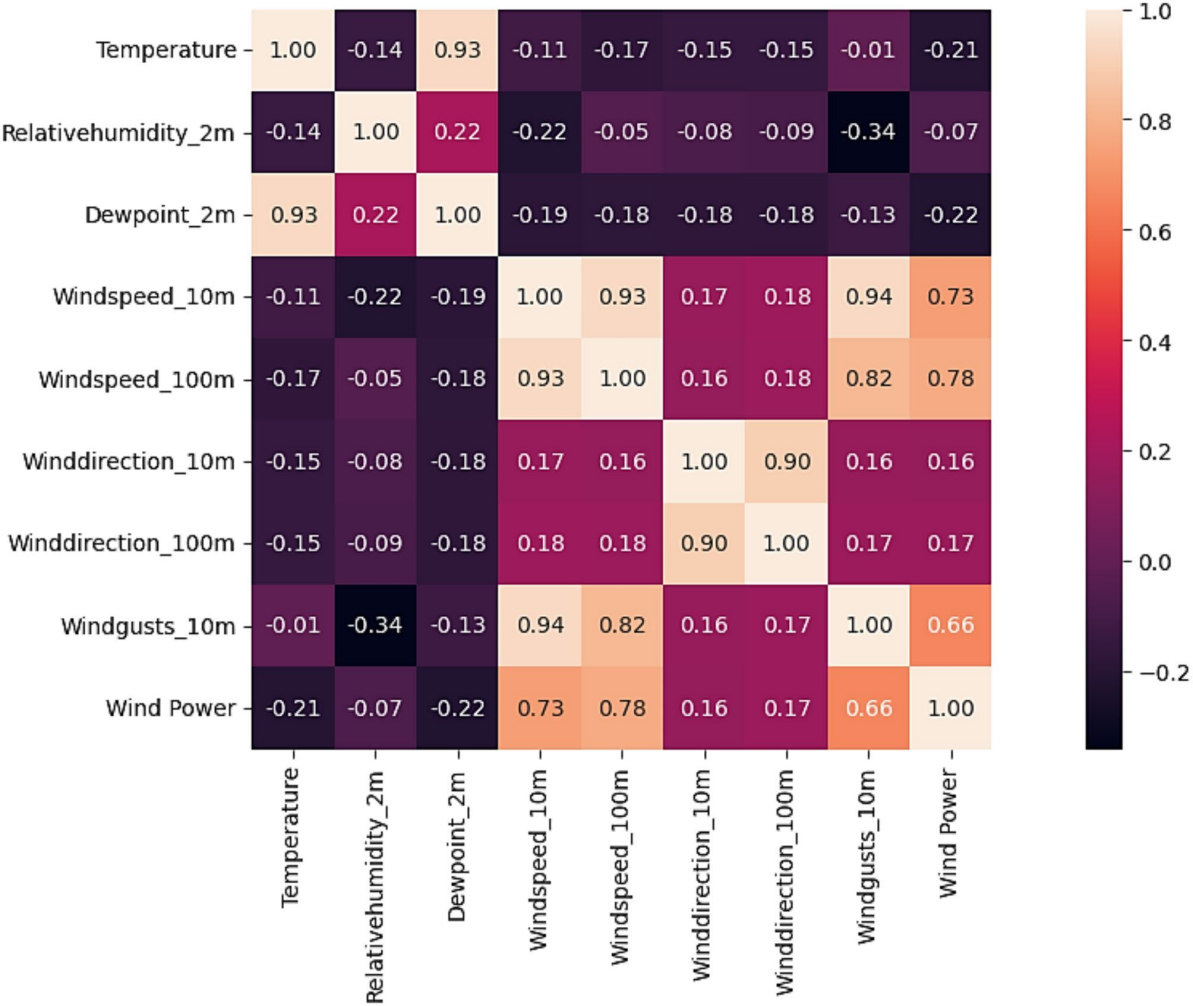
Heatmap analysis of WPG dataset feature.

**Fig. 5:**
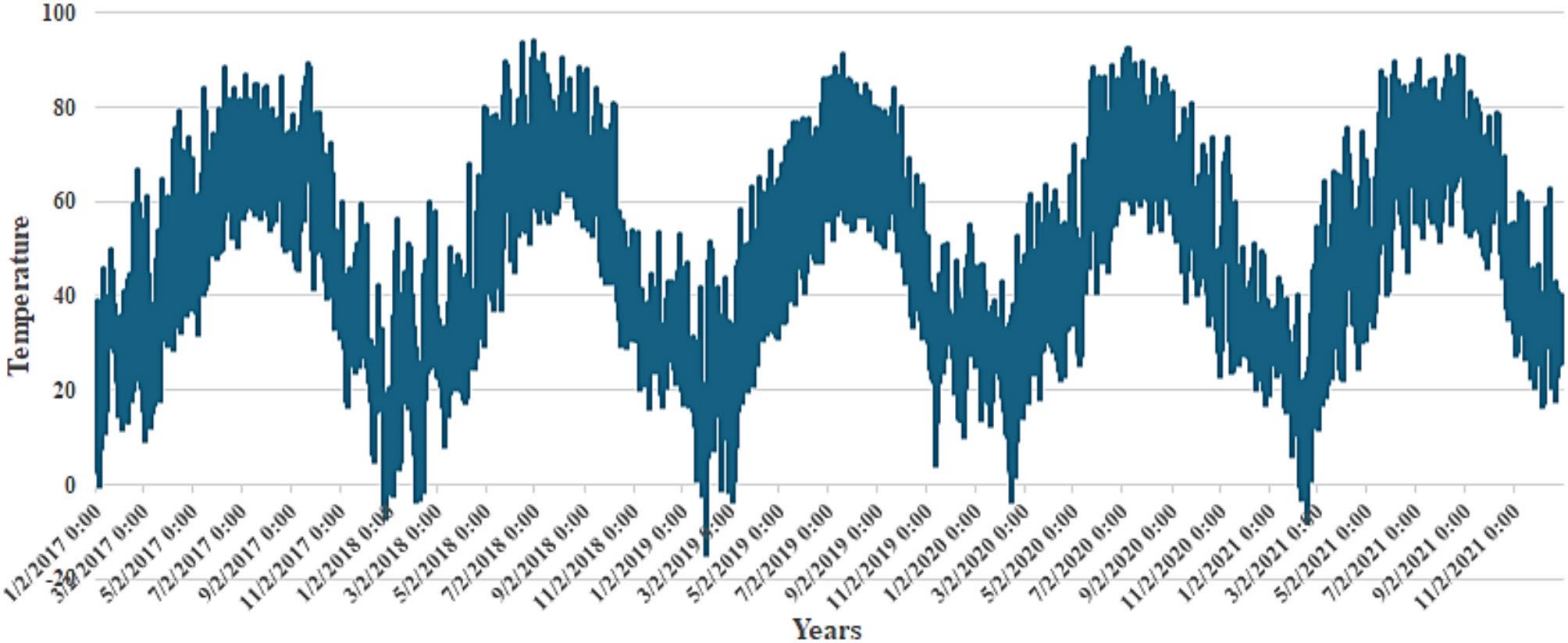
Plot between the years and the temperature.

**Fig. 6:**
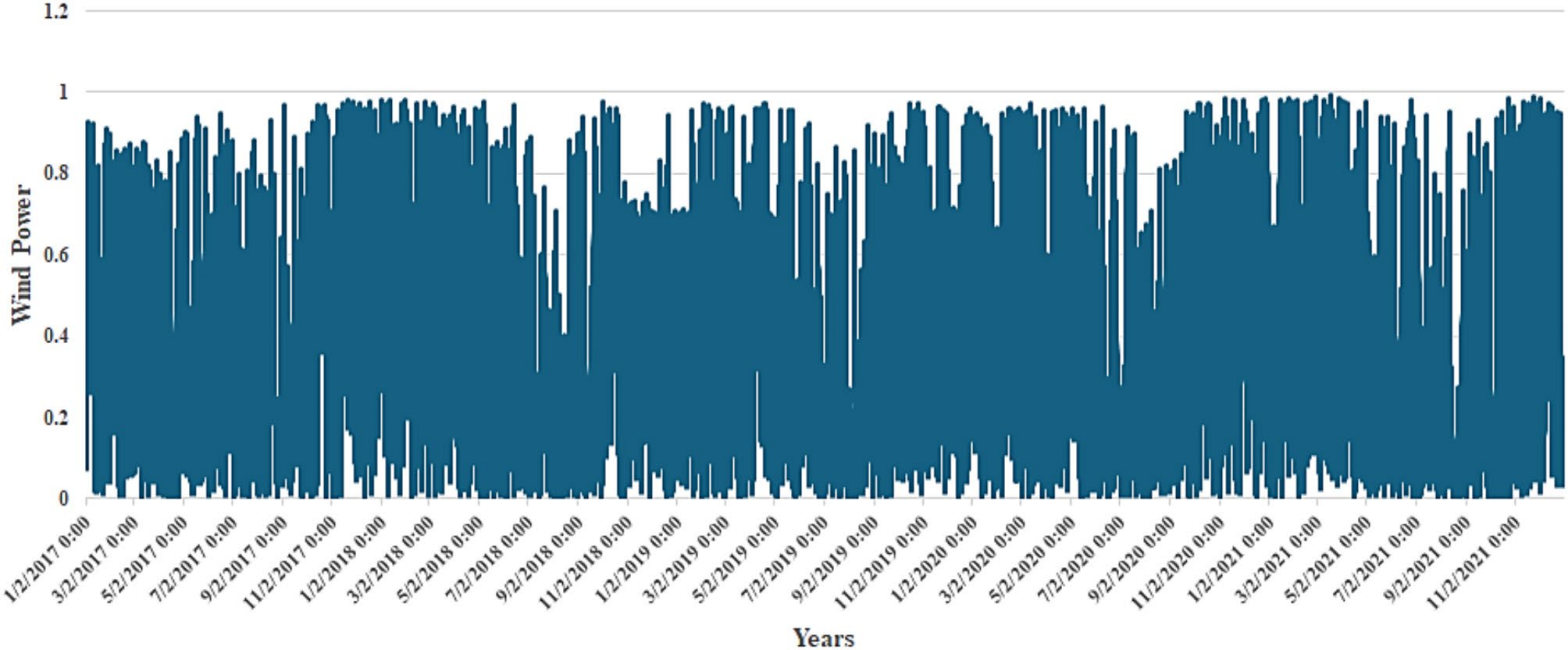
Plot between the years and wind power.

Figure 4 shows the heatmap analysis of the dataset features, which visualizes the relationships between different attributes and helps identify patterns in exploratory data analysis. Figure 5 shows the plot of temperature against the years, while Fig. 6 shows the plot of wind power against the years.

### Z-score normalization

Normalization is an important preprocessing technique for machine learning, especially for datasets with features that have varying scales^28^. Normalization transforms data values into a standard range, usually between 0 and 1. This technique ensures that all features contribute equally to the model, preventing large-scale features from dominating the learning process^29^. The mathematical formula for Z-score normalization is given by Eq. 1:

1 $$\:Z=\frac{x-min\left(x\right)}{x-max\left(x\right)}$$

where, in Eq. 1, $\:\:Z$is the transformed (normalized) data, $\:x$ stands for the original input value from the dataset, $\:min\left(x\right)$ and $\:max\left(x\right)$ are the minimum and maximum values for the provided input dataset.

### Convolutional neural network

A Convolutional Neural Network (CNN) is a deep learning model that excels at processing structured data, particularly for tasks that require spatial feature extraction. It is employed across a variety of fields and domains^30,31^. The CNN model architecture is able to learn spatial hierarchies of features applied on new data through backpropagation by employing multiple building blocks^31^:

1. **Convolutional Layers**: These layers are the essential building blocks of a CNN. They use a series of learnable filters, also known as kernels, that slide over the input data to conduct convolution operations. Several equal-sized filters are applied, and each filter is utilized to recognize a certain pattern such as edges. The convolutional operation captures the spatial hierarchies within the data via the local connectivity pattern between neurons of adjacent layers, essentially reducing the parameter space compared to fully connected layers.
2. **Activation Function**: The Rectified Linear Unit (ReLU) is a most used activation function that converts all negative values to zero and keeps all positive values. ReLU helps improve training by enabling faster convergence in deep convolutional neural networks.
3. **Pooling Layers**: Pooling, also known as downsampling, is an important step in CNNs that reduces the spatial size of feature maps. Max pooling, the most common method, selects the maximum value from each patch. This reduces computational complexity, decreases the number of parameters, and lowers the dimensionality of the feature space.
4. **Fully Connected Layers**: In a neural network, fully connected layers follow convolutional and pooling layers to execute higher-level reasoning. By connecting each neuron to the preceding layer, they combine localized features into a global representation that captures interactions between distant features.
5. **Output Layer**: The output layer of a CNN is the final layer that generates the prediction output. The Softmax activation function is frequently used to convert raw scores into probabilities and make the results sound.

### ResNet50

ResNet50 is a deep neural network with 50 layers, including convolutional, batch normalization, ReLU activation, and fully connected layers^32^. It uses residual blocks with shortcut connections to solve the vanishing gradient. These shortcut connections help the network learn more effectively, even with hundreds of layers^33^.

### Long short-term memory

Long Short-Term Memory (LSTM) is an extension of Recurrent Neural Networks (RNNs) that learns long-term dependencies in the input data and addresses the vanishing gradient problem^34^. The LSTM consists of three gates: Forget gate: A sigmoid activation function is used by the forget gate in an LSTM network to determine which data should be removed from the cell’s memory^34^. The current input and the hidden state are taken into consideration while making this choice. The output of the forget gate ranging between 0 and 1, where values close to 0 indicate forgetting and values close to 1 indicate retaining information. The input gate in an LSTM decides if new information should be stored in its memory. It consists of two layers: a sigmoid layer that selects which information to update, and a tanh activation that generates vector of candidate values to be added to memory. On the other hand, the output gate applies a sigmoid function to decide which parts of the LSTM’s memory contribute to the output. After that, a tanh function is used to scale these selected values to a range between –1 and 1^35^.

### The proposed CNN-ResNet50-LSTM model

This study presents an enhanced deep learning framework for forecasting climate change based on two key factors: temperature and wind power. The proposed CNN–ResNet50–LSTM model integrates the strengths of three components CNN for extracting detailed spatial patterns, ResNet50 for advanced deep feature learning, and LSTM for processing sequential data.

**Fig. 7:**
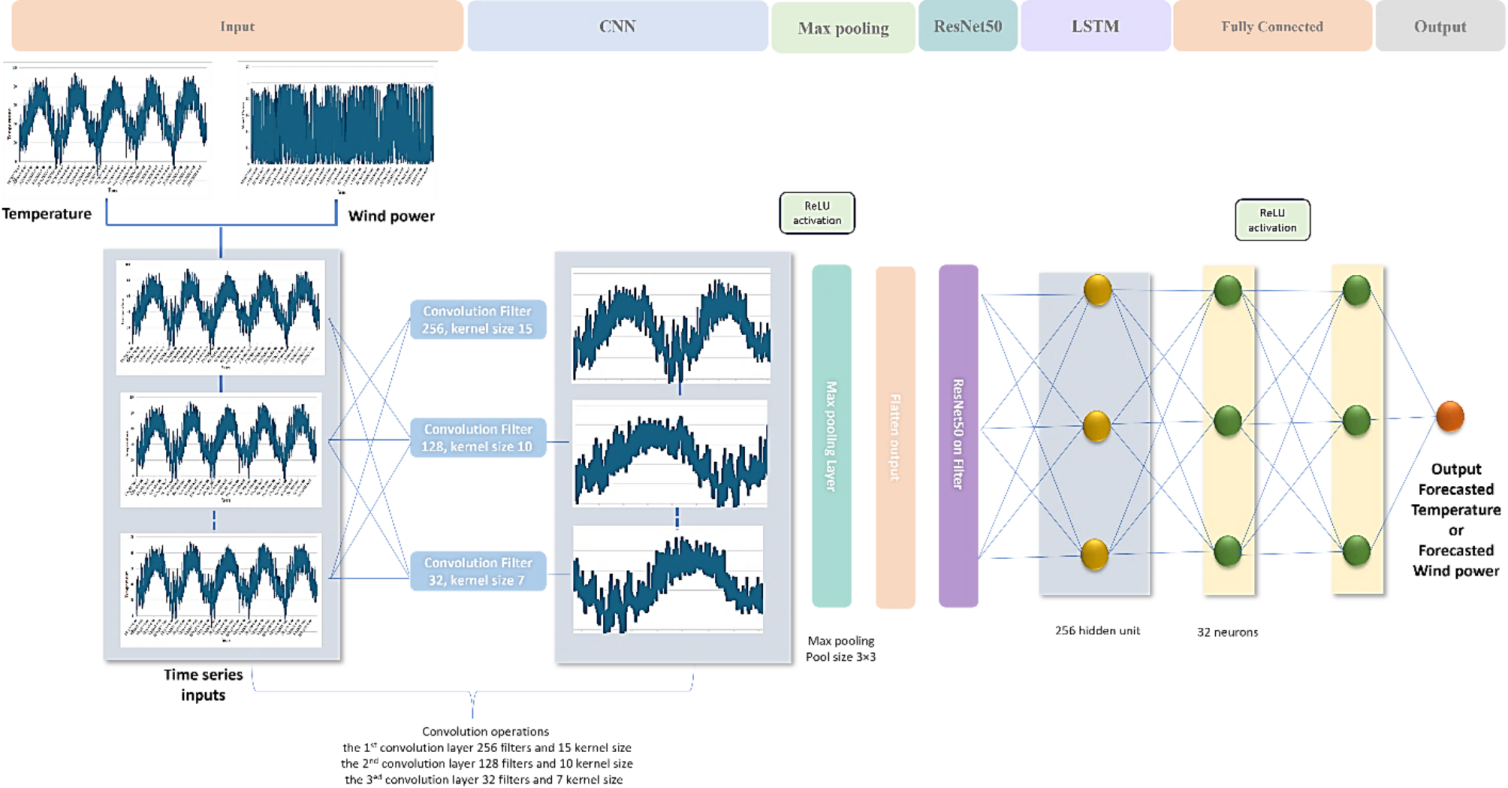
Architecture of CNN-ResNet50-LSTM model.

The architecture of the proposed CNN-ResNet50-LSTM model is shown in Fig. 7 and discussed in the following steps:

1. **Input Layer**: Input vectors of length 9, shaped as (1, 9, 1), enabling multivariate input processing.
2. **Convolutional Layers**: Three layers with ReLU activation: **1st:** 256 filters, kernel size 15 **2nd:** 128 filters, kernel size 10** 3rd:** 32 filters, kernel size 7. These extract hierarchical features and reduce overfitting.
3. **Max Pooling Layer**: 3×3 pooling reduces spatial dimensions and highlights key features.
4. **ResNet50 Layer**: Processes feature maps using residual blocks for deep feature learning and stability. Pretrained weights improve generalization.
5. **LSTM Layer**: 256 hidden units model temporal dependencies and sequence patterns.
6. **Fully Connected Layer**: A dense layer with 32 ReLU-activated neurons combines features and prevents overfitting.
7. **Output Layer**: A single neuron with linear activation outputs continuous forecasts (i.e. temperature or wind speed).

The CNN-ResNet50-LSTM model performance is largely influenced by several hyperparameters, some of which include learning rate, batch size, and number of epochs. A batch size of 256 allows for efficient training with a balance between convergence speed and memory usage. At each iteration, the learning rate of 0.01 will be used to determine the step size for weight adjustment. The Adam optimizer, which is effective in adaptive learning rates, is used to improve the training process’ efficiency. Finally, the model is trained over 50 epochs giving the model enough iterations to learn and fine-tune its parameters.

Figure 7 demonstrates the architecture of CNN-ResNet50-LSTM model. The pseudocode for CNN-ResNet50-LSTM model is demonstrated in Algorithm 1.

**Algorithm 1:**
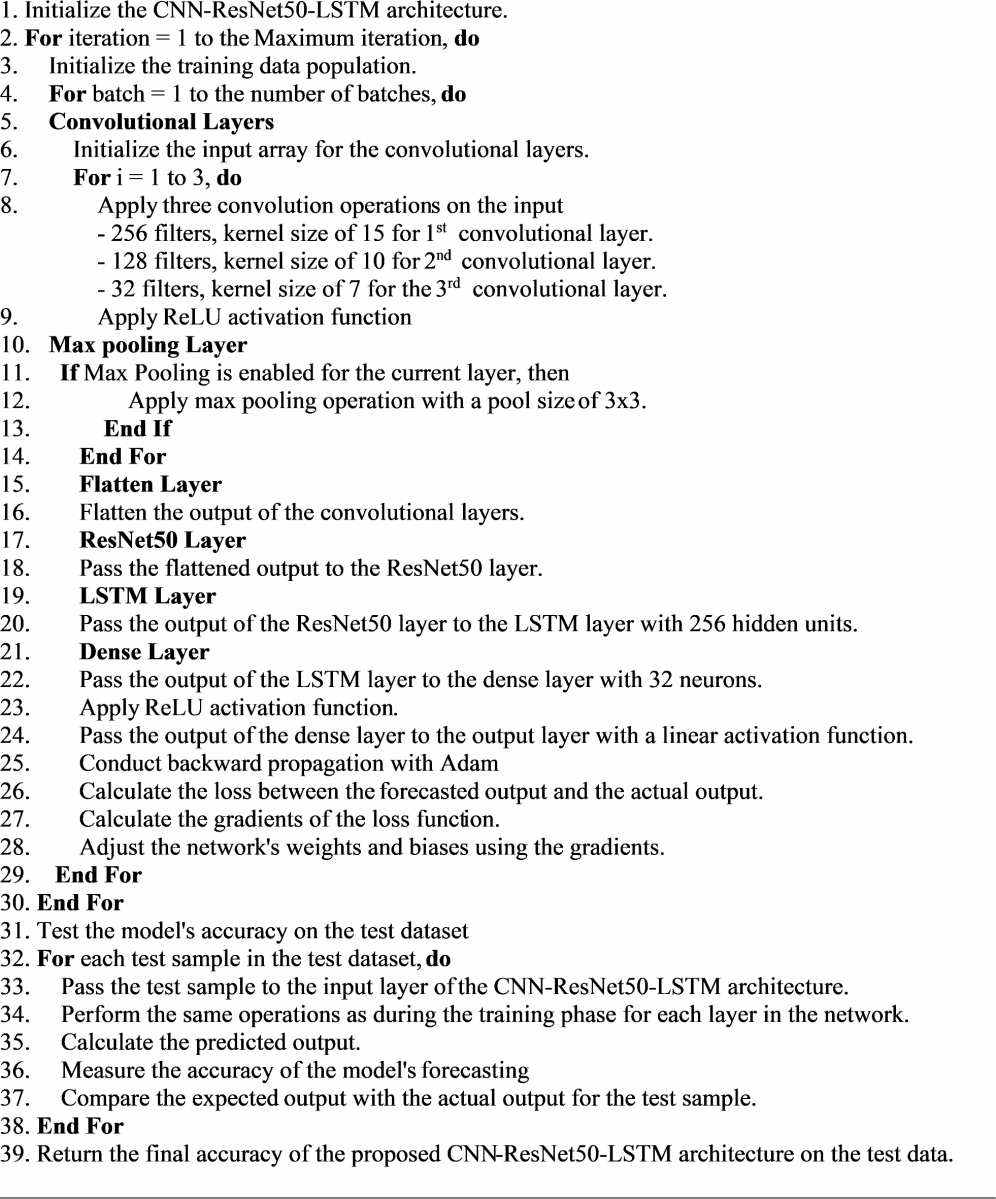
Pseudocode for CNN-ResNet50-LSTM model.

### Individual machine learning regression models

#### Kernel ridge regressor (KRR)

The Kernel Ridge Regressor (KRR) is a mix of ridge regression and kernel methods that was constructed to tackle regression problems requiring the capture of non-linear relationships in the data^36^. It uses kernel functions to map the input into a higher-dimensional feature, allowing complex patterns that linear models cannot capture to be identified. This makes KRR particularly effective for applications in which nonlinearity is prevalent, enhancing the capacity of the model to understand intricate data structures.

The fundamental mechanism of the KRR framework is based on kernel functions, or the “kernel trick,” which enables KRR to operate in an infinite-dimensional space and makes modeling extremely complex relationships simple by implicitly transforming the input data into a high-dimensional space without explicitly providing the new coordinates of the data in that space. Polynomial kernels and the Radial Basis Function (RBF) are two frequently utilized functions in kernels^37^.

#### Decision tree regressor (DTR)

Decision nodes create decisions with multiple branches, whereas leaf nodes produce outcomes with no additional branches^38^.

#### Extra trees regressor (ETR)

The Extra Trees Regressor (ETR) is an ensemble model that predicts continuous values. It generates multiple decision trees and aggregates results from multiple decision trees to output predictions^39^. Unlike other models, ETR randomly selects split points for each feature rather than finding the best splits. This helps reduce overfitting and improves generalization. ETR performs well with large datasets and is effective at modeling complex data relationships^40^.

#### Stochastic gradient descent regressor (SGDR)

Stochastic Gradient Descent Regressor (SGDR) is a machine learning model applied for regression tasks. It is called stochastic because it evaluates the gradients using randomly selected samples^41^. SGDR can be applied to various models, including logistic regression, linear regression, and neural networks^42^. 

#### Dummy regressor (DR)

The Dummy Regressor is a simple model used to set a baseline for evaluating the performance of other regression models. It does not learn any patterns from the data. Instead, it makes predictions using predefined rules based on a chosen strategy^43^. One common strategy is to always predict the mean value of the target variable from the training data^44^.

### Computational complexity

Table 5 demonstrates the computational complexity for the proposed CNN-ResNet50-LSTM model and the traditional regression models, DR, KRR, DTR, ETR, and SGDR.

**Table 5:** Computational complexity for the models used in this study.

Model	Computational complexity
CNN	$\:O\left({n}^{2}.{k}^{2}.c\right)$, $\:n$ is the input size, $\:k$ is kernel size, and $\:c$ is the number of filters.
ResNet50	$\:O({n}^{2}.d)$, $\:n$ is the input size and $\:d$ is the number of layers.
LSTM	$\:O\left({T.h}^{2}\right)$, $\:T$ is sequence length and $\:h$ is the number of hidden units.
DR	$\:O\left(1\right)$
KRR	$\:O\left({n}^{3}\right)$, $\:n$ is the number of training samples.
DTR	$\:O\left(n\text{log}n\right)$, $\:n$ is the number of training samples.
ETR	$\:O\left(n\text{log}n\right)$, $\:n$ is the number of training samples.
SGDR	$\:O(n.d)$, $\:n$ is the number of training samples, $\:d$ is the number of features.

Table 6 compares the training time and inference time for the proposed CNN-ResNet50-LSTM model and the traditional regression models, DR, KRR, DTR, ETR, and SGDR. As seen in Table 6, CNN-ResNet50-LSTM is the fastest model in both training and inference, making it ideal for real-time applications.

**Table 6:** Training time and inference time comparison.

Model	Training time (s)	Inference time (ms)
CNN-ResNet50-LSTM	1	0.1
DR	500	12
KRR	20	5
DTR	10	1.5
ETR	15	2
SGDR	8	1

### Evaluation metrics

Several evaluation metrics are used for measuring how well the proposed CNN-ResNet50-LSTM model performs compared to other models. These metrics help us understand the accuracy and reliability of predictions in different ways. The metrics include: These metrics include: Mean Squared Error (MSE), Mean Absolute Error (MAE), Median Absolute Error (MedAE), Root Mean Squared Error (RMSE), R-squared (R²), Root Mean Squared Relative Error (RMSRE), Mean Squared Relative Error (MSRE), Mean Absolute Relative Error (MARE), and Root Mean Squared Percentage Error (RMSPE). The definitions and explanations of these metrics are presented in Eqs. (2–10):

2 $$\:MSE=\frac{1}{n}\sum\:_{i=1}^{n}{({Act}_{i}-{pre}_{i})}^{2}$$

3 $$\:MAE=\frac{1}{n}\sum\:_{i=1}^{n}\left|{Act}_{i}-{pre}_{i}\right|$$

4 $$\:MedAE=median\left(\left|{pre}_{i}-{Act}_{1}\right|,\dots\:\dots\:,\left|{pre}_{i}-{Act}_{i}\right|\right)$$

5 $$\:RMSE=\sqrt{\frac{1}{n}\sum\:_{i=1}^{n}{({Act}_{i}-{pre}_{i})}^{2}}$$

6 $$\:{R}^{2}=1-\:\frac{{\sum\:}_{i=1}^{n}{\left({Act}_{i}-{pre}_{i}\right)}^{2}}{{\sum\:}_{i=1}^{n}{\left(\left({\sum\:}_{i=1}^{n}{Act}_{i}\right)-{Act}_{i}\right)}^{2}}$$

7 $$\:RMSRE=\sqrt{\frac{1}{n}\sum\:_{i=1}^{n}{\left(\frac{{Act}_{i}-{pre}_{i}}{{Act}_{i}}\right)}^{2}}$$

8 $$\:MSRE=\frac{1}{n}\sum\:_{i=1}^{n}{\left(\frac{{Act}_{i}-{pre}_{i}}{{Act}_{i}}\right)}^{2}$$

9 $$\:MARE=\frac{1}{n}\sum\:_{i=1}^{n}\left|\frac{{Act}_{i}-{pre}_{i}}{{Act}_{i}}\right|$$

10 $$\:RMSPE=\sqrt{\frac{1}{n}\sum\:_{i=1}^{n}{\left(\frac{{Act}_{i}-{pre}_{i}}{{Act}_{i}}\times\:100\right)}^{2}}$$

where in Eqs. (2–6), *n* denotes the dataset sample size, and $\:{\:Act}_{i}$, $\:{pre}_{i}$ represent the $\:{i}^{th}$ actual and forecasted values, respectively.

## Results and discussion

We conducted our experiments using Jupyter Notebook version 7.2.1. The experiments were carried out on a Windows 10 PC equipped with an Intel Core i7 processor, 32GB of RAM, and an Nvidia RTX 2080 GPU. For model development and training, we used deep learning frameworks such as Keras and TensorFlow. Additional Python libraries NumPy, Pandas, Matplotlib, and Scikit-learn were used for data preprocessing, analysis, and visualization. In this study, we introduced an improved deep learning model called CNN-ResNet50-LSTM to forecast climate change based on two key factors: temperature and wind power. We compared the performance of the proposed model with five other regression models: KRR, DTR, ETR, SGDR, DR. The evaluation was based on several performance metrics, including Mean Squared Error (MSE), Mean Absolute Error (MAE), Median Absolute Error (MedAE), Root Mean Squared Error (RMSE), and R-squared (${\text{R}}^{2}$). Experimental results show that the proposed CNN-ResNet50-LSTM model outperforms the five individual regression models in the two factors: temperature and wind power. Table 7 shows the hyperparameter values for each regression model utilized in this study.

**Table 7:** Hyperparameters for the five traditional machine learning regression models.

Models	Hyperparameters
ETR	N_estimators = 150, max_depth = 30.
SGDR	Alpha = 0.001, fit_intercept = true.
DTR	Max_depth = 100, criterion = “squared_error”.
KRR	Alpha = 1, kernel = “linear”.
DR	Strategy = “mean”.

In this paper, we used the grid search approach to experiment with different values for key hyperparameters. Table 8 demonstrates the range of the hyperparameters tested during model tuning using the grid search approach.

**Table 8:** Hyperparameters tested range for CNN-ResNet50-LSTM model.

Hyperparameter	Hyperparameters Range
Batch size	{32, 64, 128, 256}
Learning rate	{0.001, 0.005, 0.01}
Number of LSTM units	{64, 128, 256}
Number of CNN filters	{32, 64, 128, 256, 512}
Kernel sizes	{3, 5, 7, 15}
Dropout rate	{0.2, 0.3, 0.5}
Number of epochs	{20, 50, 100}

Table 9 demonstrates the tuned hyperparameters for CNN-ResNet50-LSTM model using the grid search approach.

**Table 9:** Best hyperparameters for CNN-ResNet50-LSTM model.

Hyperparameter	Best Values
Batch size	256
Learning rate	0.01 (with Adam optimizer)
Number of LSTM units	256
Number of CNN filters	256 (first layer), 128 (second layer), 32 (third layer)
Kernel sizes	15, 10, 7 for the three CNN layers
Dropout rate	0.3
Number of epochs	50

Table 10 shows the performance of the proposed CNN-ResNet50-LSTM model and five traditional machine learning regression models (ETR, SGDR, DTR, KRR and DR) in forecasting temperature and wind power in Scada, SA, and WPG datasets. We used evaluation metrics outlined in Eqs. (2–6) to assess their performance. The proposed CNN-ResNet50-LSTM model outperformed other models in forecasting temperature and wind power across three datasets: Scada, SA, and WPG. For Scada dataset, the proposed CNN-ResNet50-LSTM model achieves the lowest MSE (0.0302), MAE (0.1005), MedAE (0.0876), RMSE (0.1737), and the highest $\:{\text{R}}^{2}$ (98.84%). On the other hand, the DR model performed the worst, with higher MSE (0.1062), MAE (0.8142), MedAE (0.4881), RMSE (0.3258), and the lowest $\:{\text{R}}^{2}$ (92.05%). In SA dataset, the CNN-ResNet50-LSTM model achieved the best results with the lowest MSE (0.0399), MAE (0.1099), MedAE (0.0983), RMSE (0.1997), and the highest $\:{\text{R}}^{2}$ (99.01%). On the other hand, the KRR and DR model performed the worst, with MSE (0.1251), MAE (0.7894), MedAE (0.7551), RMSE (0.3536), and the lowest $\:{\text{R}}^{2}$ (91.03%) in DR and MSE (0.1036), MAE (0.6142), MedAE (0.5092), RMSE (0.3218), and the lowest $\:{\text{R}}^{2}$ (92.15%) in KRR. Finally, in the WPG dataset our proposed CNN-ResNet50-LSTM model came out on top in forecasting temperature, achieving the best results with the lowest MSE (0.0087), MAE (0.0824), MedAE (0.0713), RMSE (0.0932), and the highest $\:{\text{R}}^{2}$ (98.58%). On the other hand, the DR model performed the worst, with higher MSE (0.2684), MAE (1.8629), MedAE (1.0591), RMSE (0.5180), and the lowest $\:{\text{R}}^{2}$ (89.83%). Likewise, the proposed CNN-ResNet50-LSTM model achieved the best results in forecasting wind power in WPG dataset with the lowest MSE (0.0103), MAE (0.0814), MedAE (0.0686), RMSE (0.1015), and the highest $\:{\text{R}}^{2}$ (98.53%). On the other hand, the DR model performed the worst, with higher MSE (0.3681), MAE (0.9494), MedAE (0.8692), RMSE (0.6067), and the lowest $\:{\text{R}}^{2}$ (88.94%). These results show that our proposed CNN-ResNet50-LSTM model is much better at forecasting wind power than the other five traditional regression models. These results show that our proposed CNN-ResNet50-LSTM model is much better at forecasting temperature than the other five traditional regression models.

**Table 10:** Performance of the proposed CNN-ResNet50-LSTM model and the individual regression models for temperature and wind forecasting in Scada, SA, and WPG datasets using MSE, MAE, medae, RMSE, and R^2^ metrics.

	Model	MSE	MAE	MedAE	RMSE	*R* ^2^
Scada dataset	CNN-ResNet50-LSTM	0.0302	0.1005	0.0876	0.1737	98.84%
Wind power forecasting	ETR	0.0415	0.1121	0.0945	0.2037	97.14%
	SGDR	0.0463	0.1187	0.0991	0.2151	96.47%
	DTR	0.0574	0.1562	0.1054	0.2395	95.05%
	KRR	0.0786	0.3948	0.1936	0.2803	93.96%
	DR	0.1062	0.8142	0.4881	0.3258	92.05%
	CNN-ResNet50-LSTM	0.0399	0.1099	0.0983	0.1997	99.01%
SA dataset	ETR	0.0447	0.1194	0.1052	0.2114	97.23%
Temperature forecasting	SGDR	0.0563	0.1352	0.1217	0.2372	96.05%
	DTR	0.0874	0.4265	0.3141	0.2956	93.72%
	KRR	0.1036	0.6142	0.5092	0.3218	92.15%
	DR	0.1251	0.7894	0.7551	0.3536	91.03%
	CNN-ResNet50-LSTM	0.0087	0.0824	0.0713	0.0932	98.58%
WPG dataset	ETR	0.0253	0.1038	0.0946	0.1590	95.86%
Temperature forecasting	SGDR	0.0362	0.2681	0.2037	0.1902	94.92%
	DTR	0.0636	0.5827	0.4946	0.2521	93.29%
	KRR	0.0985	0.9494	0.8925	0.3138	91.32%
	DR	0.2684	1.8629	1.0591	0.5180	89.83%
	CNN-ResNet50-LSTM	0.0103	0.0814	0.0686	0.1015	98.53%
WPG dataset	ETR	0.0358	0.1493	0.0953	0.1892	95.73%
Wind power forecasting	SGDR	0.0661	0.3572	0.2539	0.2570	94.85%
	DTR	0.0748	0.4754	0.3246	0.2734	92.87%
	KRR	0.0957	0.6843	0.5463	0.3093	90.74%
	DR	0.3681	0.9494	0.8692	0.6067	88.94%

Table 11 shows the performance of the proposed CNN-ResNet50-LSTM model and five traditional machine learning regression models (ETR, SGDR, DTR, KRR and DR) in forecasting temperature and wind power for Scada, SA, and WPG datasets. We used evaluation metrics outlined in Eqs. (7–10) to assess their performance. The proposed CNN-ResNet50-LSTM model outperformed other models in forecasting temperature and wind power across three datasets: Scada, SA, and WPG. The proposed CNN-ResNet50-LSTM model achieves the lowest MSRE, MARE, RMSRE, and the highest RMSPE for the three datasets. On the other hand, the DR model performed the worst, with the highest MSRE, MARE, RMSRE, and the lowest RMSPE for the three datasets.

**Table 11:** Performance of the proposed CNN-ResNet50-LSTM model and the individual regression models for temperature and wind power forecasting in Scada, SA, and WPG datasets using MSRE, MARE, RMSRE, and RMSPE metrics.

	Model	MSRE	MARE	RMSRE	RMSPE
Scada dataset	CNN-ResNet50-LSTM	0.0028	0.0731	0.1463	99.08%
Wind power forecasting	ETR	0.0141	0.0847	0.1763	97.38%
	SGDR	0.0189	0.0913	0.1877	96.71%
	DTR	0.03	0.1288	0.2121	95.29%
	KRR	0.0512	0.3674	0.2529	94.20%
	DR	0.0788	0.7868	0.2984	92.29%
	CNN-ResNet50-LSTM	0.0125	0.0825	0.1723	99.25%
SA dataset	ETR	0.0173	0.092	0.184	97.47%
Temperature forecasting	SGDR	0.0289	0.1078	0.2098	96.29%
	DTR	0.06	0.3991	0.2682	93.96%
	KRR	0.0762	0.5868	0.2944	92.39%
	DR	0.0977	0.762	0.3262	91.27%
	CNN-ResNet50-LSTM	0.00596	0.055	0.0658	98.82%
WPG dataset	ETR	0.02256	0.0764	0.1316	96.10%
Temperature forecasting	SGDR	0.03346	0.2407	0.1628	95.16%
	DTR	0.06086	0.5553	0.2247	93.53%
	KRR	0.09576	0.922	0.2864	91.56%
	DR	0.26566	1.8355	0.4906	90.07%
	CNN-ResNet50-LSTM	0.00756	0.054	0.0741	98.77%
WPG dataset	ETR	0.03306	0.1219	0.1618	95.97%
Wind power forecasting	SGDR	0.06336	0.3298	0.2296	95.09%
	DTR	0.07206	0.448	0.246	93.11%
	KRR	0.09296	0.6569	0.2819	90.98%
	DR	0.36536	0.922	0.5793	89.18%

Table 12 demonstrates the performance of the proposed CNN-ResNet50-LSTM model and five advanced deep learning models (CNN, LSTM, GRU, ResNet50 and VGG19) in forecasting temperature and wind power for Scada, SA, and WPG datasets. We used evaluation metrics, namely, MSE, MAE, MedAE, and R^2^ to assess their performance. The proposed CNN-ResNet50-LSTM model outperformed other models in forecasting temperature and wind power across three datasets: Scada, SA, and WPG. The proposed CNN-ResNet50-LSTM model achieves the lowest MSE, MAE, MedAE, and the highest R^2^ for the three datasets. On the other hand, the VGG19 model performed the worst, with the highest MSE, MAE, MedAE, and the lowest R^2^ for the three datasets.

**Table 12:** Performance of the proposed CNN-ResNet50-LSTM model and advanced deep learning models for temperature and wind forecasting in Scada, SA, and WPG datasets using MSE, MAE, medae, and R^2^ metrics.

	Model	MSE	MAE	MedAE	*R* ^2^
Scada dataset	CNN-ResNet50-LSTM	0.0302	0.1005	0.0876	98.84%
Wind power forecasting	CNN	0.0223	0.0929	0.1845	97.34%
	LSTM	0.0271	0.0995	0.1959	96.67%
	GRU	0.0382	0.137	0.2203	95.25%
	ResNet50	0.0594	0.3756	0.2611	94.16%
	VGG19	0.087	0.795	0.3066	92.25%
	CNN-ResNet50-LSTM	0.0399	0.1099	0.0983	99.01%
SA dataset	CNN	0.0255	0.1002	0.1922	97.43%
Temperature forecasting	LSTM	0.0371	0.116	0.218	96.25%
	GRU	0.0682	0.4073	0.2764	93.92%
	ResNet50	0.0844	0.595	0.3026	92.35%
	VGG19	0.1059	0.7702	0.3344	91.23%
	CNN-ResNet50-LSTM	0.0087	0.0824	0.0713	98.58%
WPG dataset	CNN	0.0061	0.0846	0.1398	96.06%
Temperature forecasting	LSTM	0.017	0.2489	0.171	95.12%
	GRU	0.0444	0.5635	0.2329	93.49%
	ResNet50	0.0793	0.9302	0.2946	91.52%
	VGG19	0.2492	1.8437	0.4988	90.03%
	CNN-ResNet50-LSTM	0.0103	0.0814	0.0686	98.53%
WPG dataset	CNN	0.0166	0.1301	0.17	95.93%
Wind power forecasting	LSTM	0.0469	0.338	0.2378	95.05%
	GRU	0.0556	0.4562	0.2542	93.07%
	ResNet50	0.0765	0.6651	0.2901	90.94%
	VGG19	0.3489	0.9302	0.5875	89.14%

**Fig. 8:**
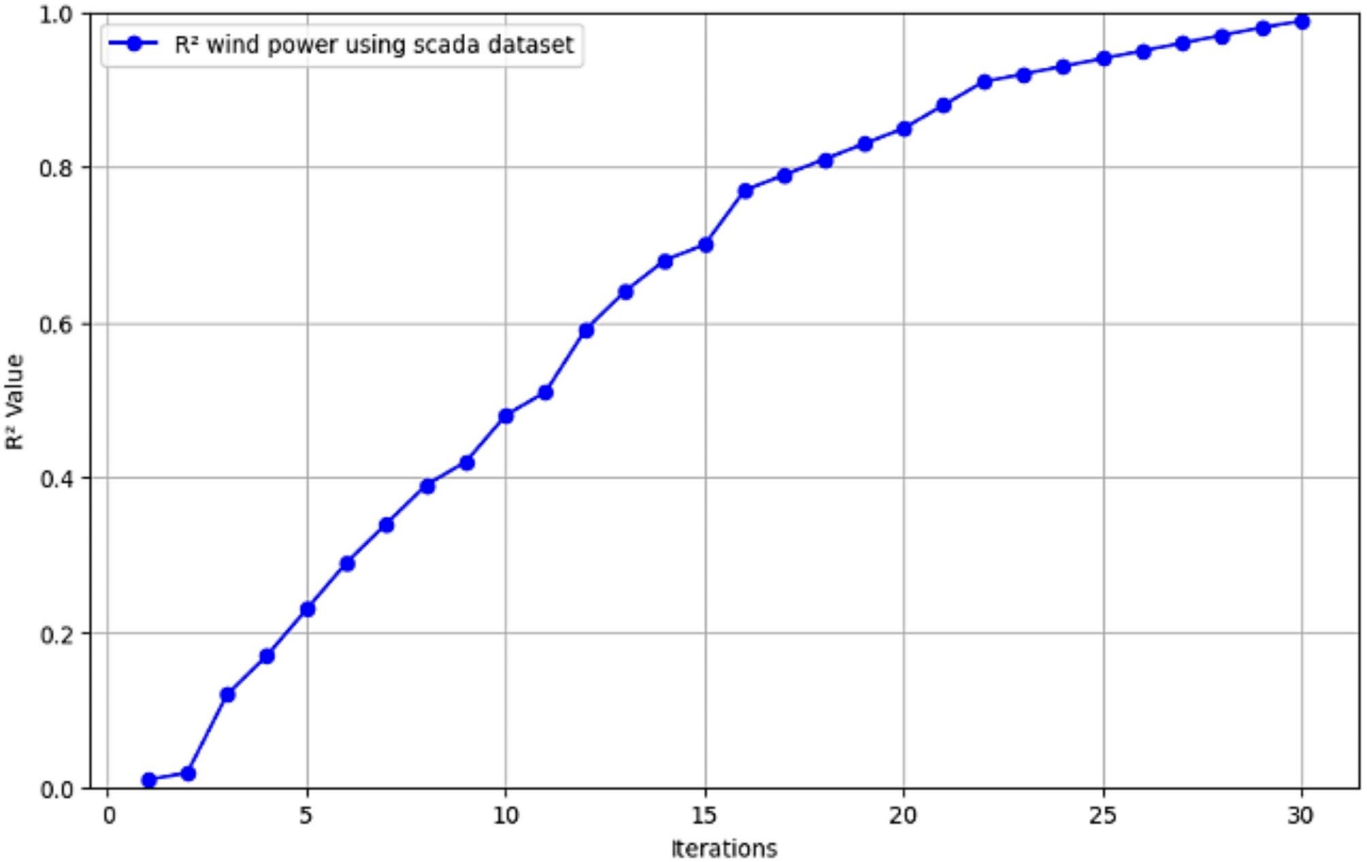
$\:{\text{R}}^{2}$ convergence curve for CNN-ResNet50-LSTM model for wind power forecasting in Scada dataset.

**Fig. 9:**
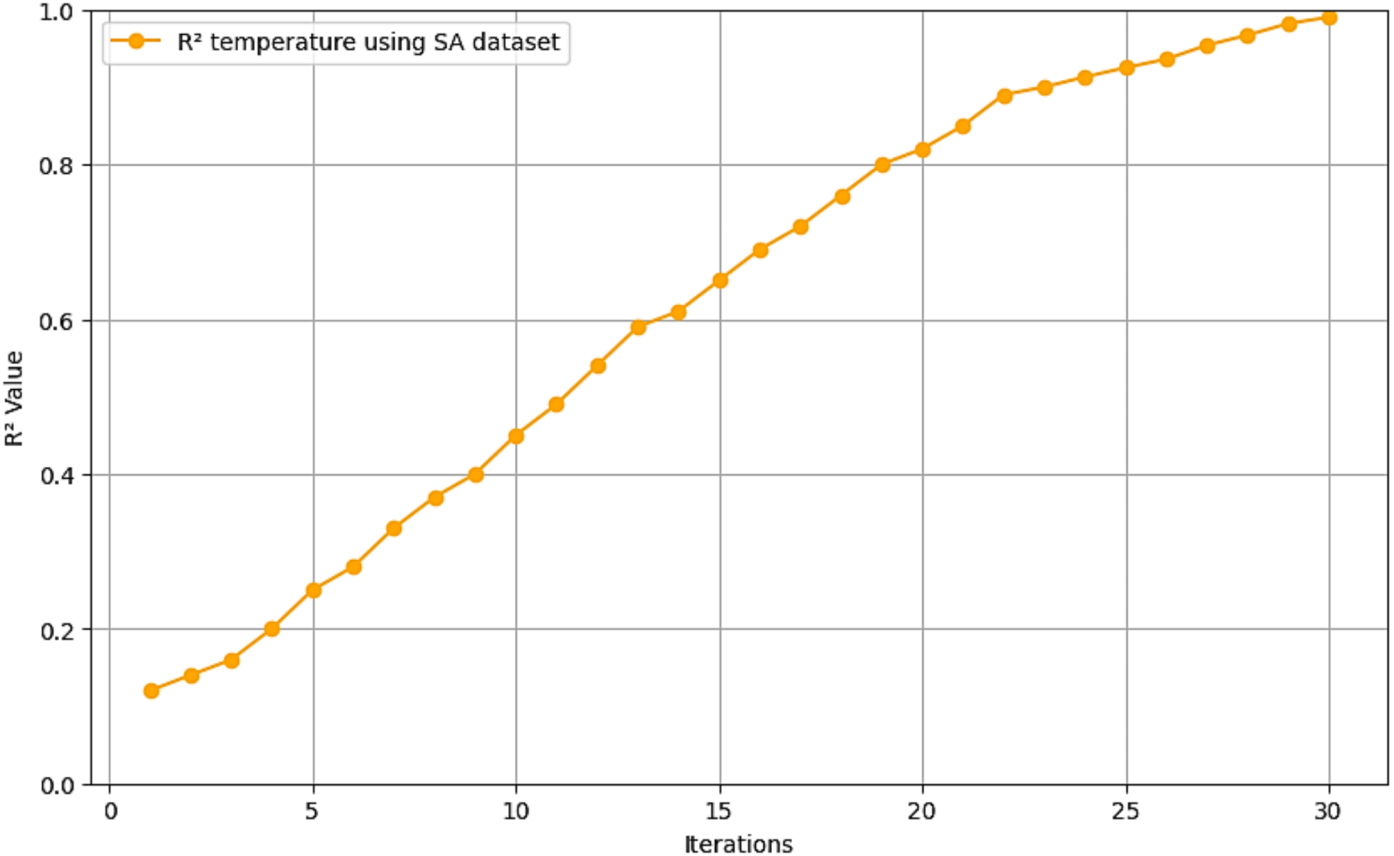
$\:{\text{R}}^{2}$ convergence curve for CNN-ResNet50-LSTM model for temperature forecasting in SA dataset.

**Fig. 10:**
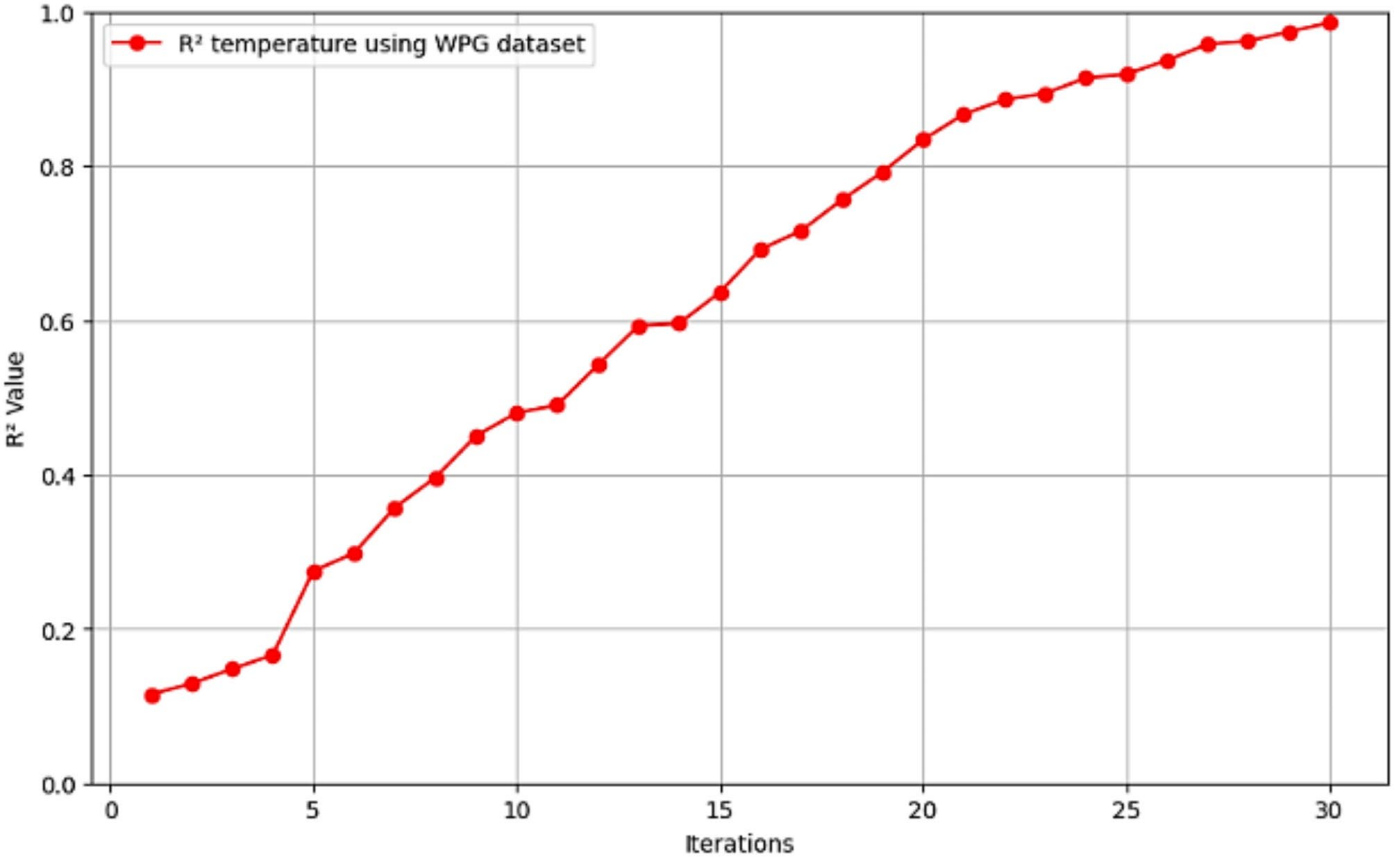
$\:{\text{R}}^{2}$ convergence curve for CNN-ResNet50-LSTM model for temperature forecasting in WPG dataset.

**Fig. 11:**
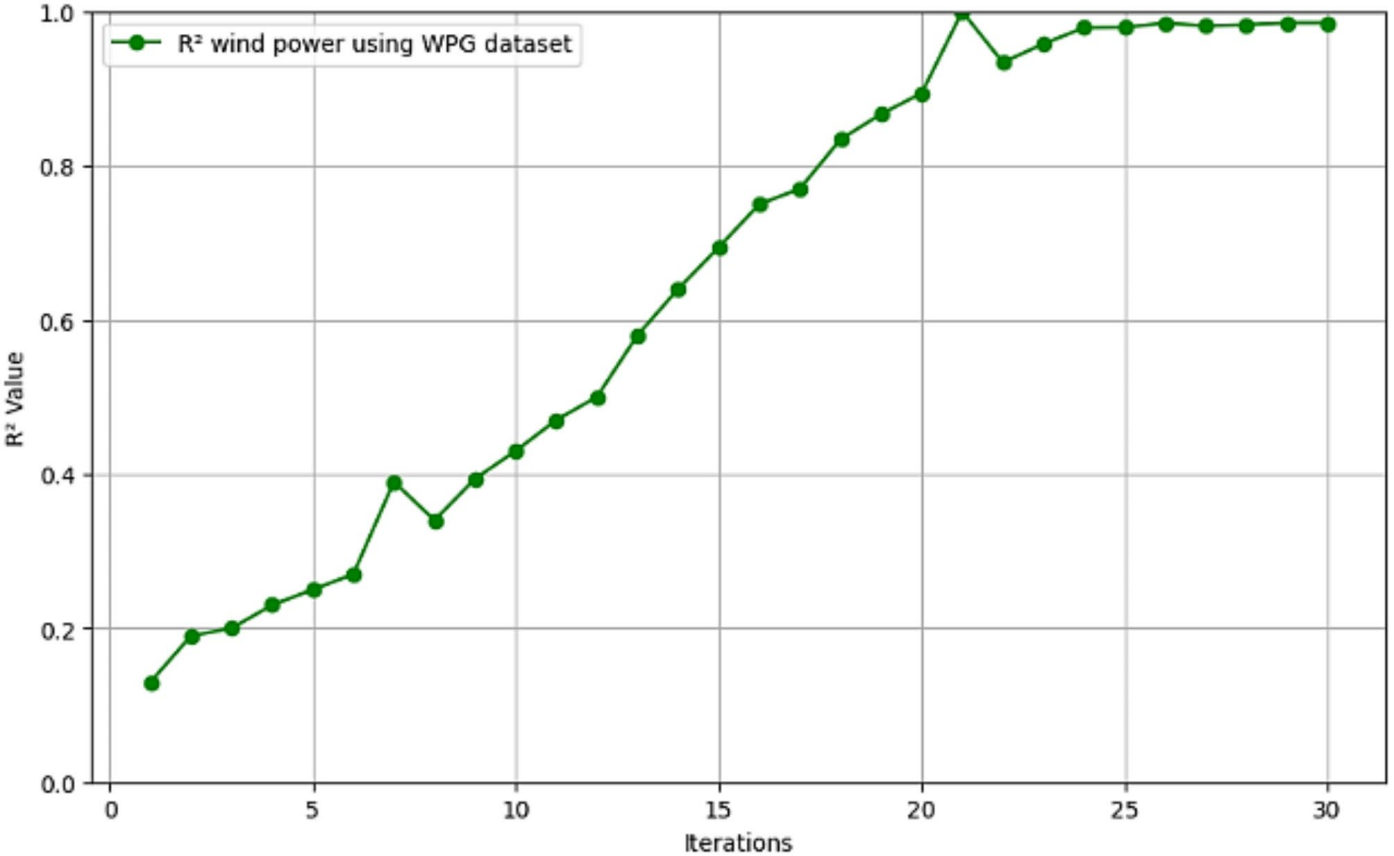
$\:{\text{R}}^{2}$ convergence curve for CNN-ResNet50-LSTM model for wind power forecasting in WPG dataset.

**Fig. 12:**
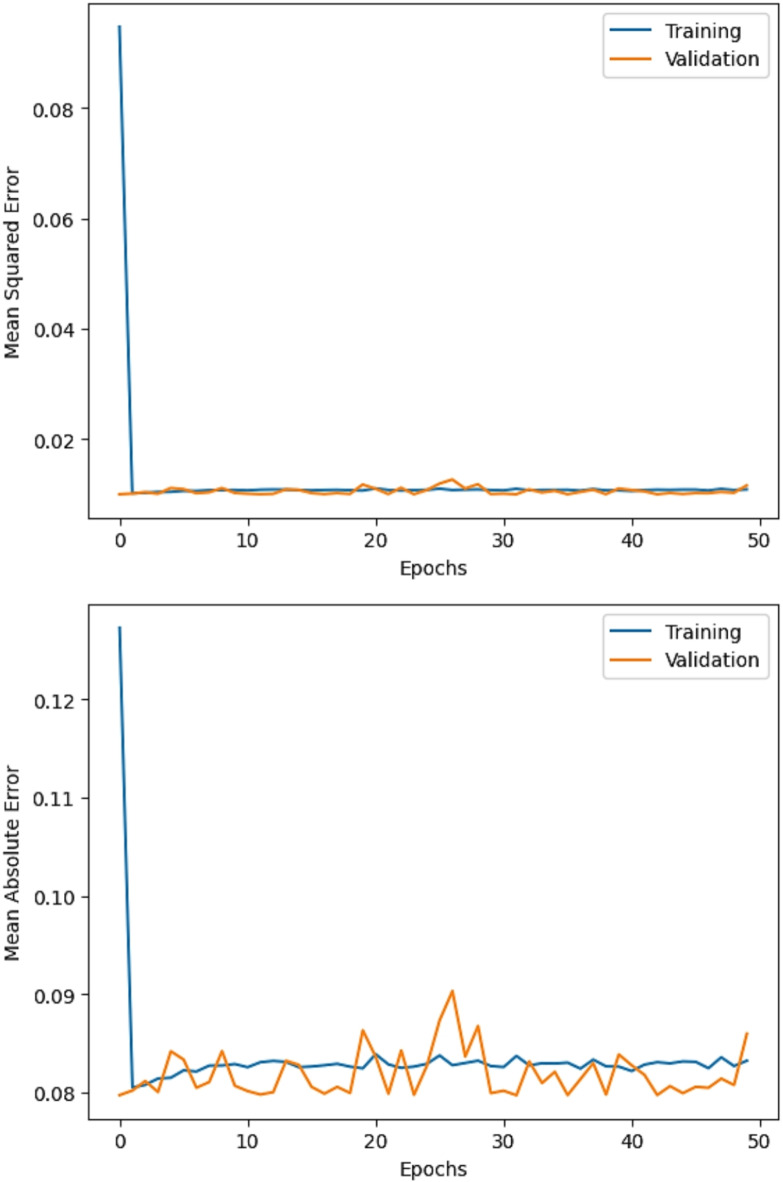
The curves of training and validation for MSE and MAE against the number of epochs using CNN-ResNet50-LSTM model for wind power forecasting in Scada dataset.

**Fig. 13:**
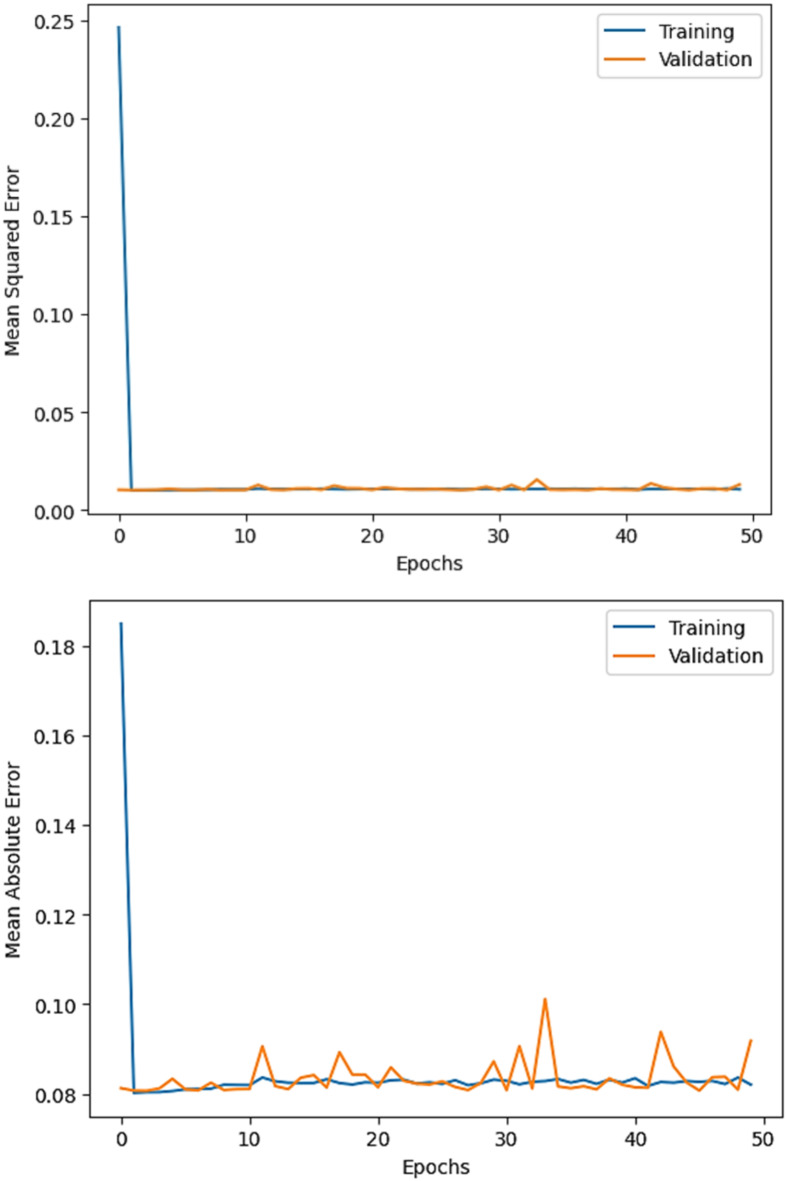
The curves of training and validation for MSE and MAE against the number of epochs using CNN-ResNet50-LSTM model for temperature forecasting in SA dataset.

**Fig. 14:**
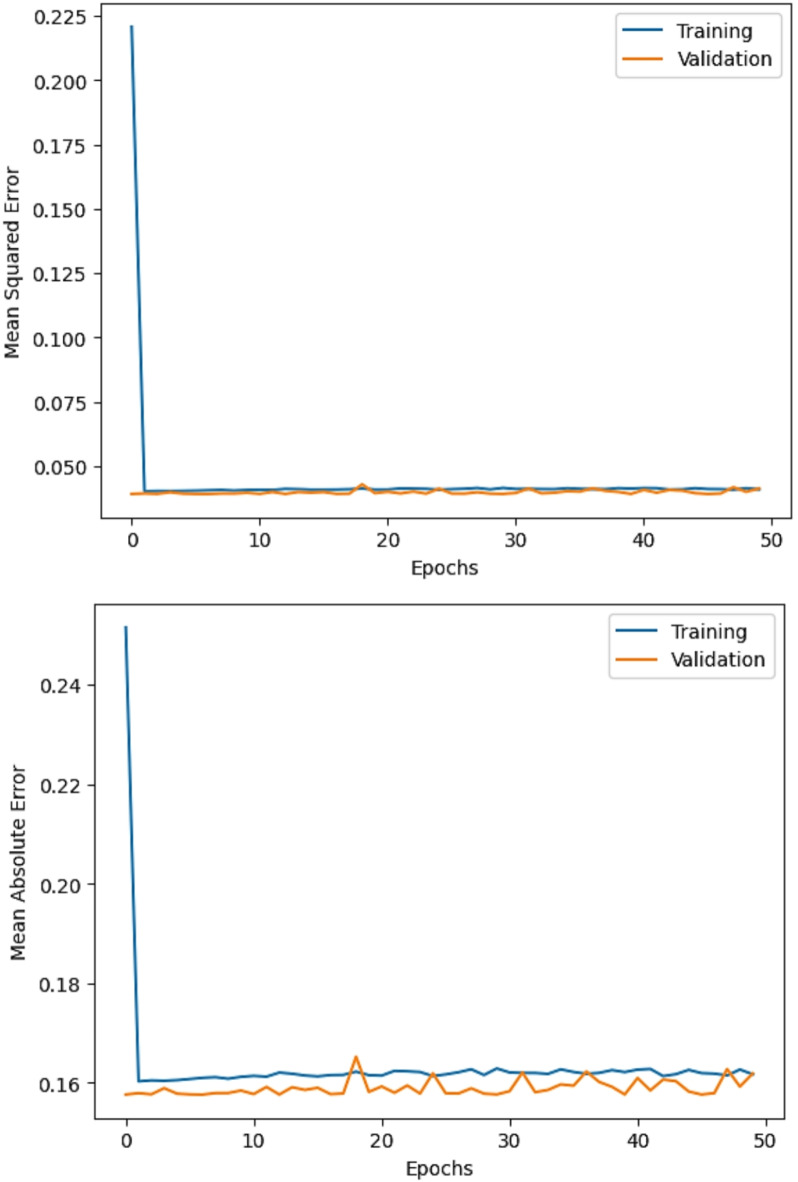
The curves of training and validation for MSE and MAE against the number of epochs using CNN-ResNet50-LSTM model for temperature forecasting in WPG dataset.

**Fig. 15:**
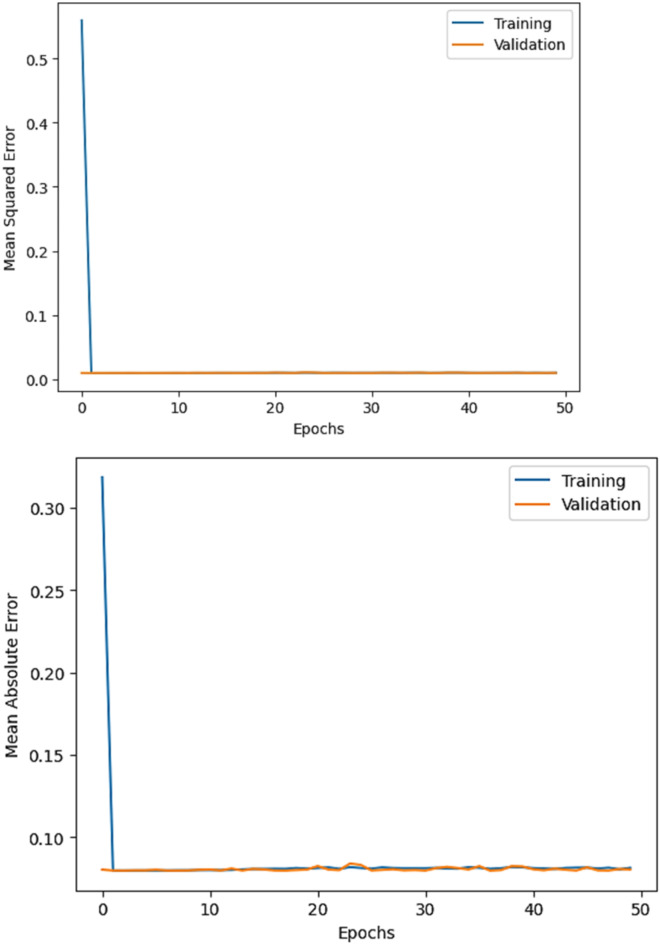
The curves of training and validation for MSE and MAE against the number of epochs using CNN-ResNet50-LSTM model for wind power forecasting in WPG dataset.

**Fig. 16:**
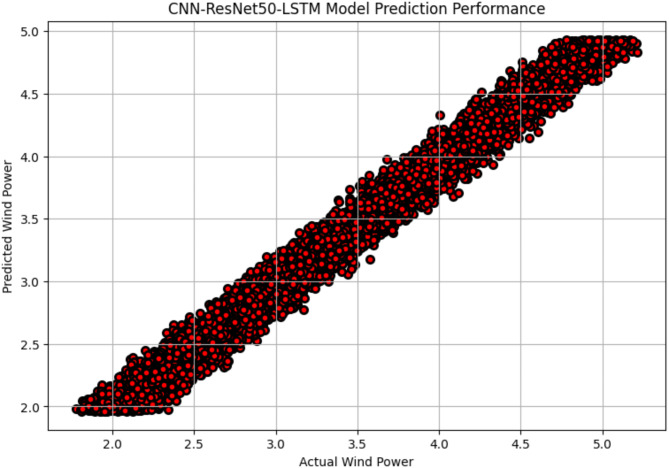
Actual wind power vs. forecasted wind power using CNN-ResNet50-LSTM forecasting model in Scada dataset.

**Fig. 17:**
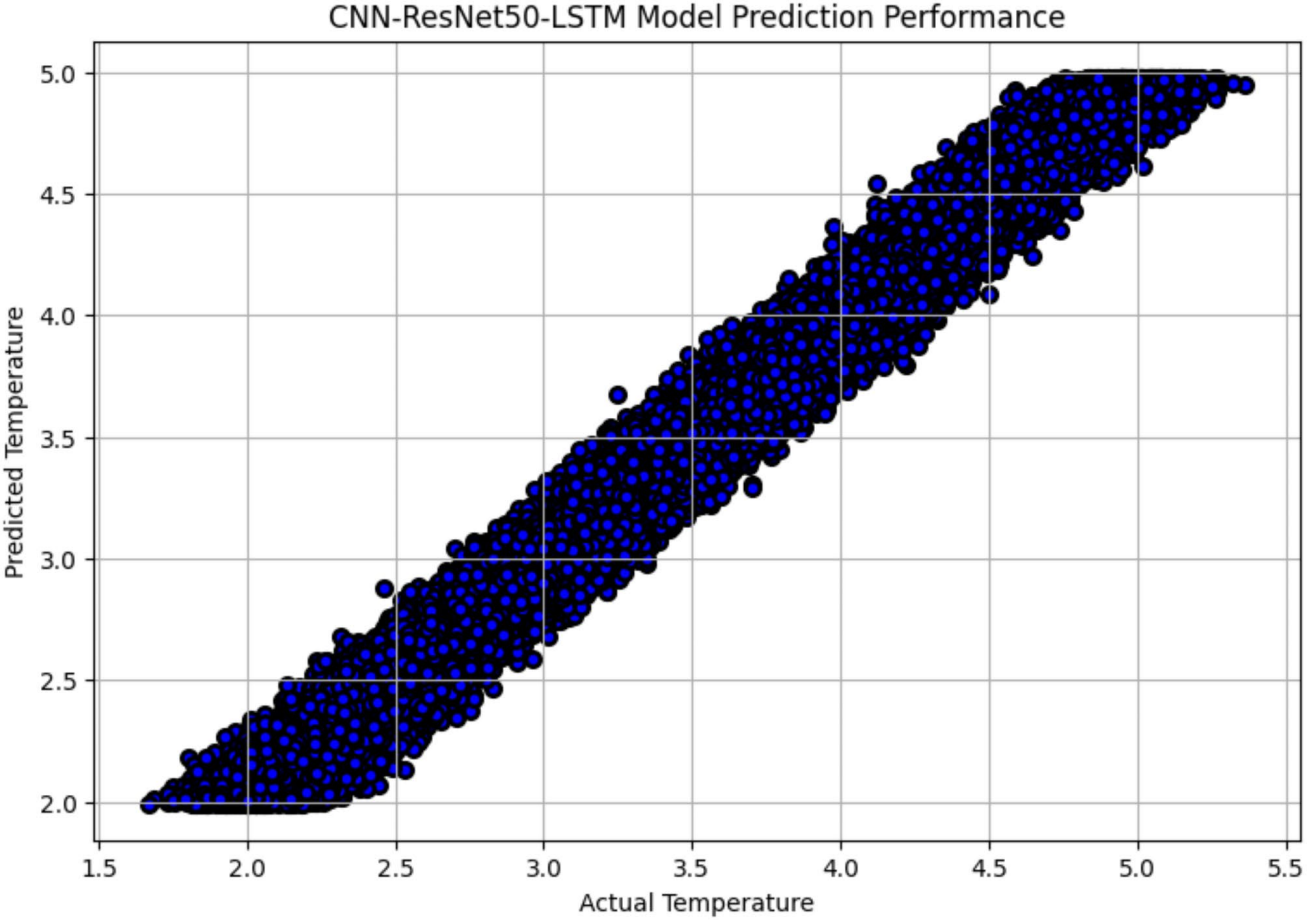
Actual temperature vs. forecasted temperature using CNN-ResNet50-LSTM forecasting model in SA dataset.

**Fig. 18:**
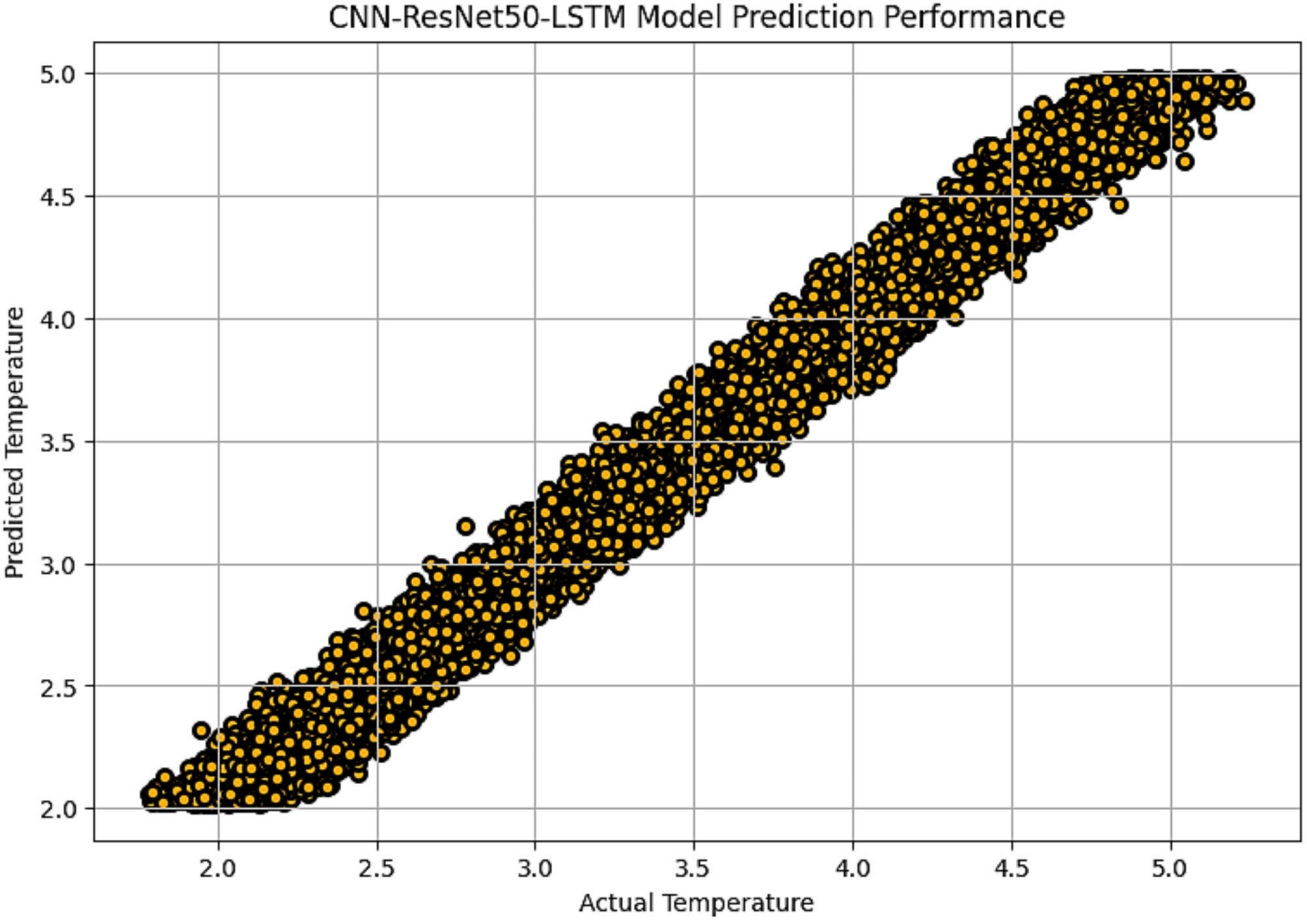
Actual temperature vs. forecasted temperature using CNN-ResNet50-LSTM forecasting model in WPG dataset.

**Fig. 19:**
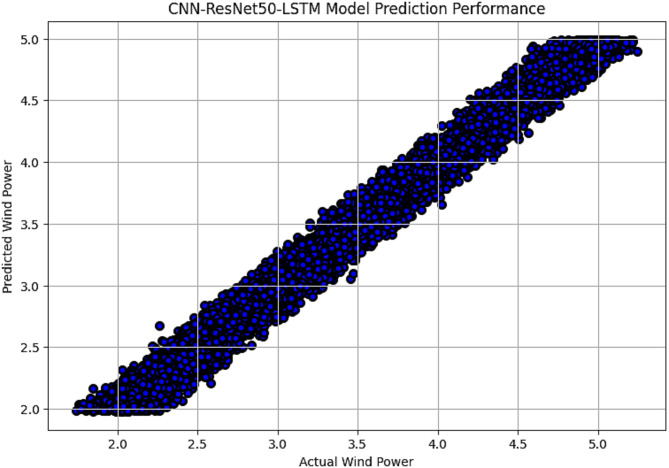
Actual wind power vs. forecasted wind power using CNN-ResNet50-LSTM forecasting model in WPG dataset.

The R^2^ convergence curves that the model produced are shown in Figs. 8, 9, 10 and 11, which provides important information about the training behavior and performance of the proposed CNN-ResNet50-LSTM model. It strengthens the study findings and allows for a more thorough understanding of the effectiveness of CNN-ResNet50-LSTM model in addressing climate change, particularly temperature and wind power. Figures 12, 13, 14 and 15 shows the training and validation curves for MSE and MAE against the number of epochs using the proposed CNN-ResNet50-LSTM model for wind power and temperature forecasting in Scada, SA, and WPG datasets. The curves indicate that the model learns and generalizes well from the training data. Figures 16, 17, 18 and 19 show a comparison of the actual temperature and wind power versus the forecasting performance for temperature and wind power using CNN-ResNet50-LSTM model for Scada, SA, and WPG datasets. The close alignment of these values shows that the model performs reliably on new, unseen data, providing accurate temperature forecasting.

**Fig. 20:**
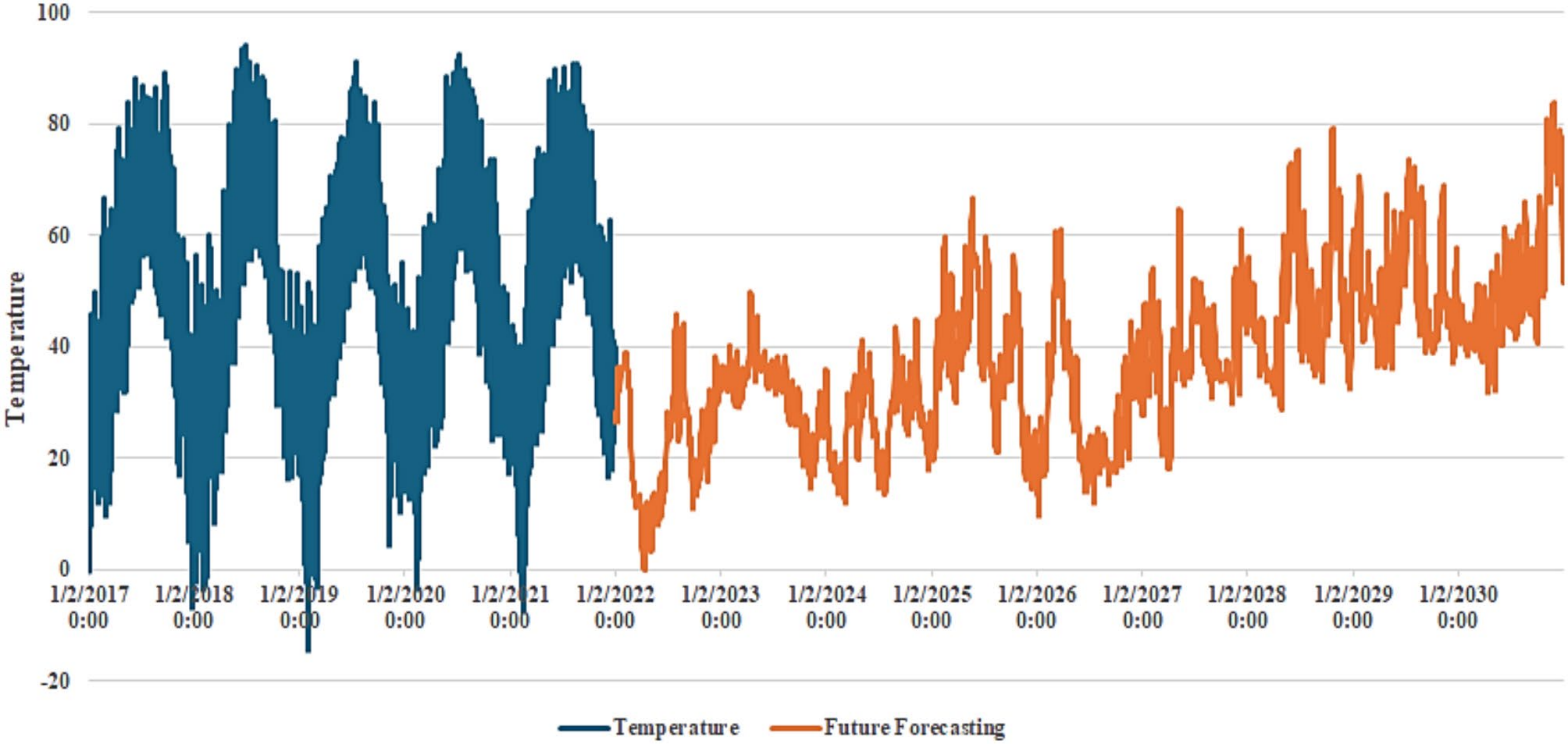
Actual temperature data (in blue) from 2017 to 2021, and the future temperature forecasting (in orange) from 2021 to 2030 using the proposed CNN-ResNet50-LSTM model.

**Fig. 21:**
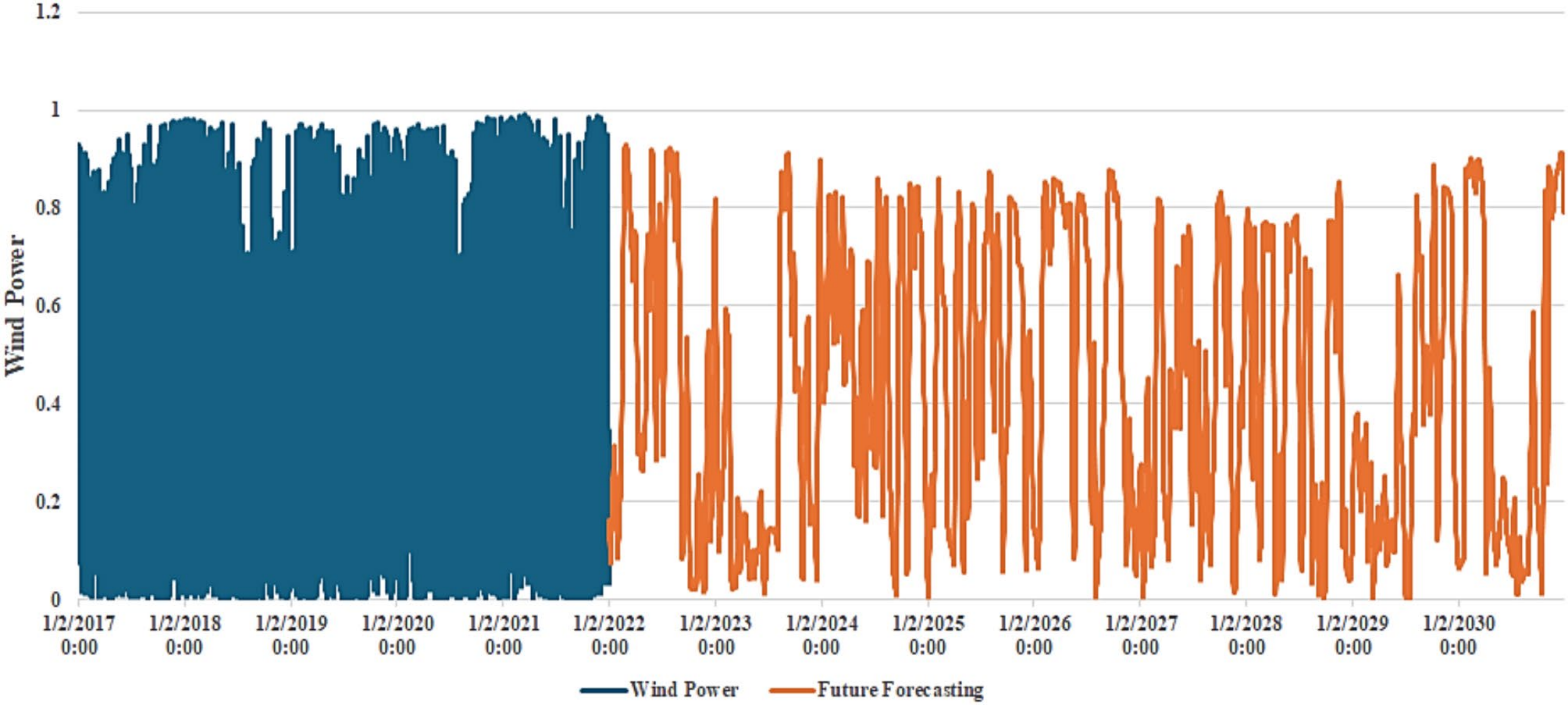
Actual wind power data (in blue) from 2017 to 2021, and the future wind power forecasting (in orange) from 2021 to 2030 using the proposed CNN-ResNet50-LSTM model.

Weather patterns are changing, and global temperatures and sea levels will continue to rise. Deep learning models can help anticipate the future of climate change. These mathematical climate change models employ historical weather data to improve the accuracy of weather forecasts. The proposed CNN-ResNet50**-**LSTM model aims to provide accurate future forecasting of temperature and wind power. Figure 20 shows actual temperature data (in blue) from 2017 to 2021, and the future temperature forecasting (in orange) from 2021 to 2030 using the proposed CNN-ResNet50-LSTM model. The blue section displays historical temperature patterns with clear seasonal variations. As we move into the orange section, the model forecasts future temperatures, capturing both short-term and long-term trends. Projections indicate a gradual increase in temperatures over the years, indicating a possible warming trend. This visualization highlights how well the proposed CNN-ResNet50-LSTM model transitions from historical data to future forecasts, demonstrating its accuracy in forecasting temperature trends through 2030. Figure 21 shows actual wind power data (in blue) from 2017 to 2021, and the future wind power forecasting (in orange) from 2021 to 2030 using the proposed CNN-ResNet50-LSTM model. The blue section displays historical wind power patterns, which remain relatively stable and high over the four-year period. As we move into the orange section, the model’s forecasts show significant fluctuations in future wind power. These forecasts capture both short-term variations and long-term trends, indicating periods of both high and low wind power availability. This visualization highlights the proposed CNN-ResNet50-LSTM model’s ability to transition smoothly from historical data to future forecasts, demonstrating its reliability in forecasting wind power trends up to the year 2030.

Forecasting the future of climate change with deep learning algorithms has major real-world implications. Here are some significant benefits of accurate future prediction:

- Accurate forecasting of global climate change can inform policymakers about potential outcomes and environmental implications, leading to more effective policies and long-term strategies.
- Climate change can lead to increased plant disease, causing significant negative impacts to plant species, production of food, and ecosystem sustainability^45^. Using deep learning algorithms helps in advance planning and developing strategies for disease management.
- Accurate forecasting of climate change factors such as temperature and wind power can predict potential extreme weather occurrences. For example, deep learning algorithms have been effectively used for flood prediction based on temperature and rainfall intensity^46^.
- Understanding associations between different climate factors and natural resources can assist in natural resource safeguarding and enhancing agriculture productivity^47^. Deep learning models can help optimize the allocation of natural resources such as water sources and food production and inform sustainable management.
- Accurate forecasting of climate change-related factors can lead to better economic planning and decision-making to foster a more sustainable economy and economic decisions. Deep learning approaches has found to be successful in forecasting renewable energy generation and electricity demand^48^.

**Fig. 22:**
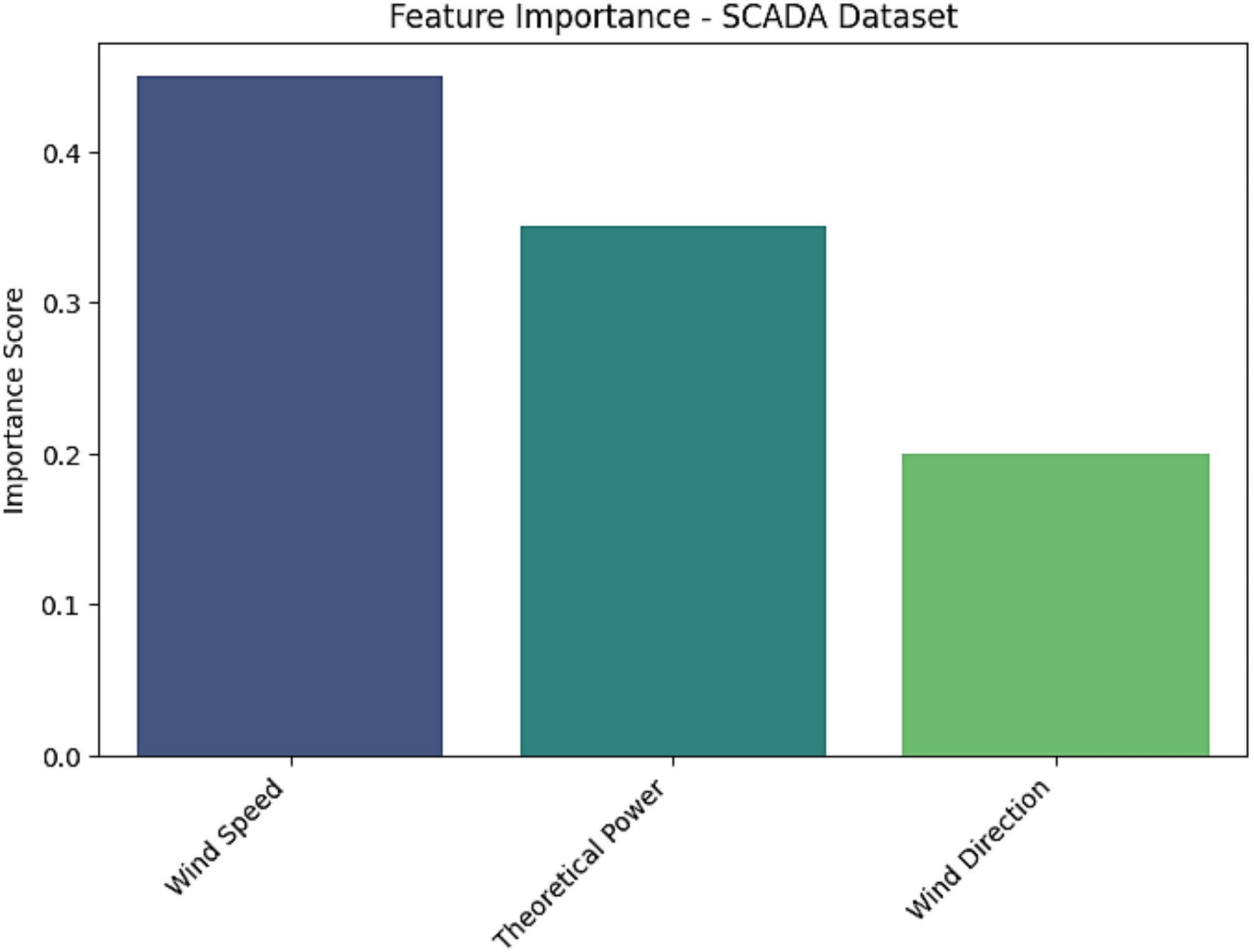
Feature importance for SCADA dataset.

**Fig. 23:**
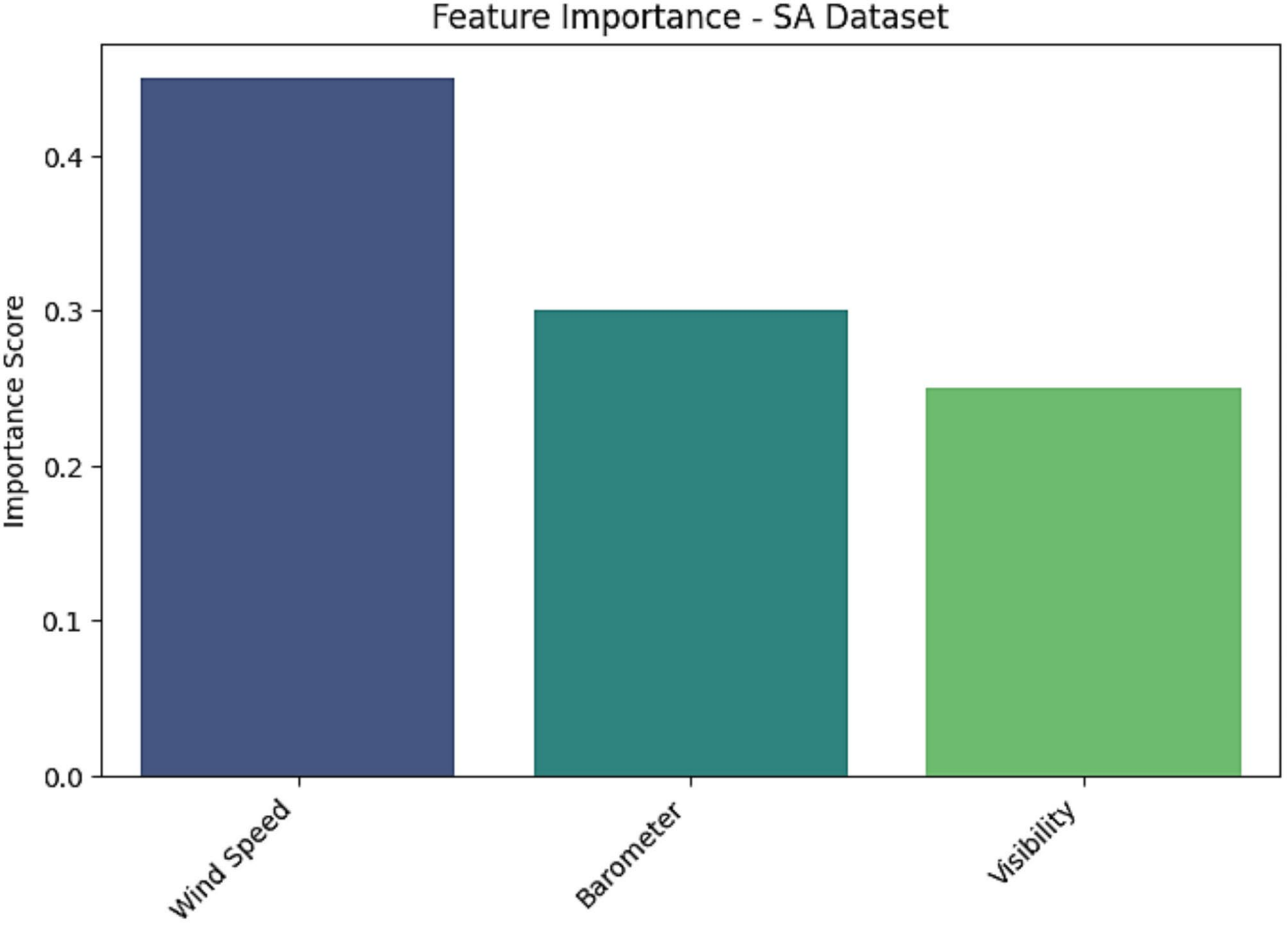
Feature importance for SA dataset.

**Fig. 24:**
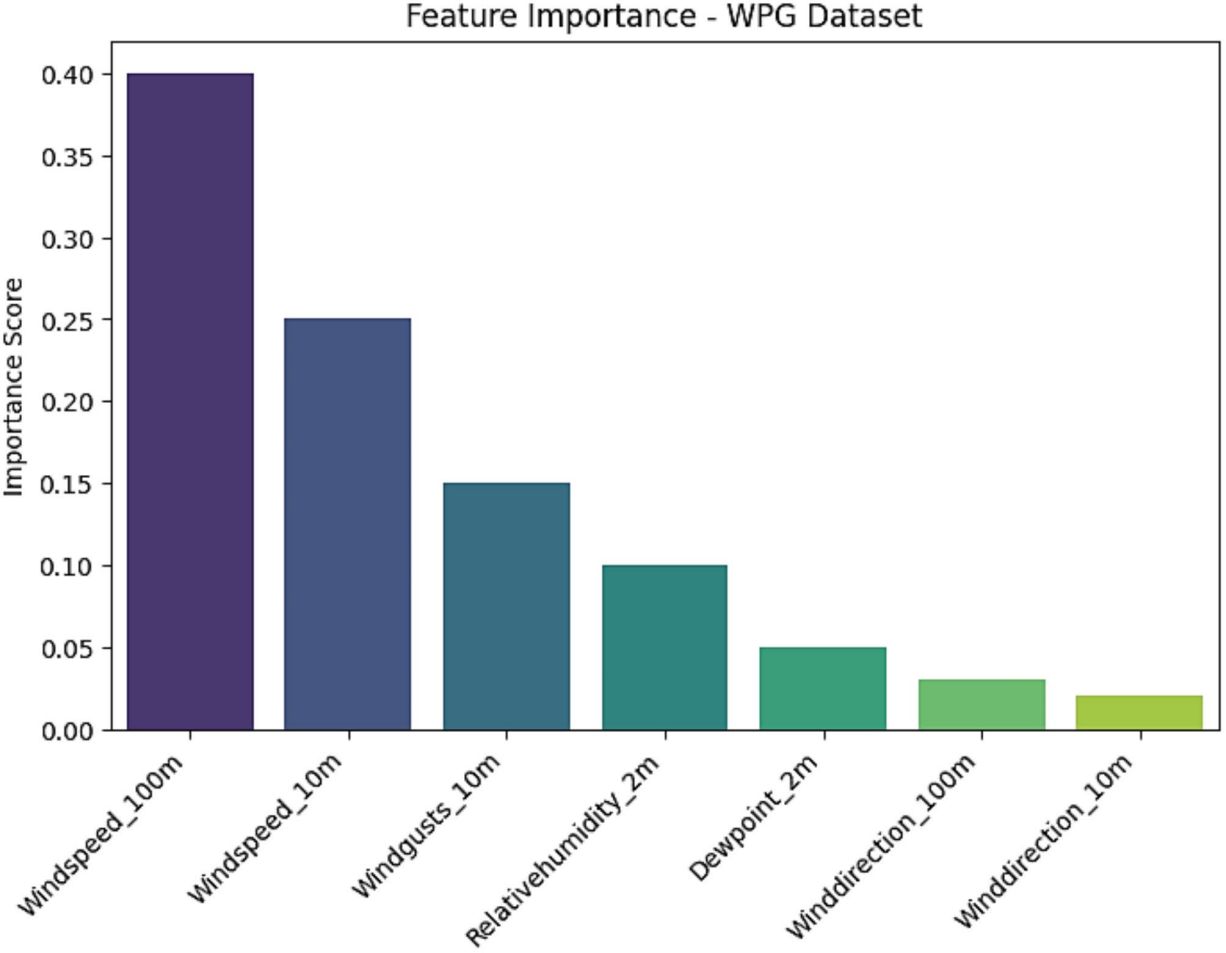
Feature importance for WPG dataset.

There is a considerable need to enhance the literature on deep learning modeling in forecasting climate change, as well as compare the performance of forecasting deep learning models. Furthermore, having abundant clean data is required for optimal outcomes of deep-learning models. In addition, the integration of many fields such as Internet of Things (IoT) and machine learning (ML) techniques are crucial to advance the domain of climate change forecasting. Figures 22, 23 and 24 demonstrate the feature importance for SCADA dataset, SA dataset, and WPG dataset.

## Sensitivity analysis

Table 13 presents the results of a sensitivity analysis conducted on the Scada dataset to evaluate how changes in key input features (Wind Speed, Wind Direction, and Theoretical Power) affect the Root Mean Squared Error (RMSE) and coefficient of determination (R^2^) for wind power forecasting. The feature column represents the input variable being perturbed. Perturbation column represents the percentage change applied to the feature values (+ 5%, -5%, + 10%, -10%). RMSE Change (%) column represents the percentage change in RMSE due to the perturbation. A higher RMSE indicates a decrease in model accuracy. R^2^ Change (%) column represents the percentage change in R^2^ due to the perturbation. A lower R^2^ means the model explains less variance in wind power predictions. Wind Speed has the strongest impact on wind power forecasting. A + 10% increase in wind speed results in a 4.3% increase in RMSE (higher error) and a 2.5% drop in R^2^, meaning the model struggles more with prediction accuracy. A -10% decrease in wind speed improves R^2^ by 2.1%, meaning predictions become slightly more accurate. Wind Direction has a moderate effect. A + 10% change in wind direction increases RMSE by 3.2%, showing that directional changes impact power generation. The R² drops by 1.9%, but the effect is smaller than for wind speed. Theoretical Power has a smaller but noticeable impact. A + 5% increase in theoretical power increases RMSE by 1.8%, while a -5% decrease slightly improves R^2^ by 0.9%. The changes in RMSE and R^2^ are relatively lower compared to Wind Speed.

**Table 13:** Sensitivity analysis for wind power forecasting (Scada dataset).

Feature	Perturbation (%)	RMSE change (%)	*R*^2^ change (%)
Wind speed	+ 5	+ 2.1	− 1.2
	− 5	− 1.8	+ 0.9
	+ 10	+ 4.3	− 2.5
	− 10	− 3.9	+ 2.1
Wind direction	+ 5	+ 1.5	− 0.8
	− 5	− 1.3	+ 0.7
	+ 10	+ 3.2	− 1.9
	− 10	− 2.9	+ 1.5
Theoretical power	+ 5	+ 1.8	− 1.0
	− 5	− 1.6	+ 0.9

Table 14 presents the results of a sensitivity analysis on the Saudi Arabia Weather History (SA) dataset, evaluating how perturbations in key features (Temperature, Wind Speed, and Barometer Pressure) affect the Root Mean Squared Error (RMSE) and coefficient of determination (R^2^) in temperature forecasting. Temperature is the most influential feature. A + 10% increase in temperature leads to a 3.8% increase in RMSE (higher prediction error) and a 2.2% decrease in R^2^, meaning the model struggles more. A -10% decrease in temperature improves R^2^ by 1.9%, suggesting that the model is highly dependent on temperature stability. Wind Speed has a strong effect on temperature forecasting. A + 10% increase in wind speed increases RMSE by 4.5%, showing that wind fluctuations significantly impact temperature predictions. The R^2^ drops by 2.6%, meaning the model becomes less reliable. A -10% decrease in wind speed improves R² by 2.3%, confirming that wind dynamics play an important role in temperature changes. Barometer Pressure has the least effect. A + 5% increase in barometer pressure only increases RMSE by 1.1%, while R^2^ decreases by 0.6%, meaning barometer pressure has a minimal impact on temperature predictions. A -5% decrease in Barometer pressure slightly improves R^2^ by 0.5%, reinforcing that pressure variations do not significantly influence temperature forecasting.

**Table 14:** Sensitivity analysis for temperature forecasting (SA dataset).

Feature	Perturbation	RMSE change (%)	*R*^2^ change (%)
Temperature	+ 5	+ 1.9	− 1.1
	− 5	− 1.6	+ 0.8
	+ 10	+ 3.8	− 2.2
	− 10	− 3.5	+ 1.9
Wind speed	+ 5	+ 2.2	− 1.3
	− 5	− 1.9	+ 1.1
	+ 10	+ 4.5	− 2.6
	− 10	− 4.1	+ 2.3
Barometer pressure	+ 5	+ 1.1	− 0.6
	− 5	− 0.9	+ 0.5

Table 15 presents the results of a sensitivity analysis on the Wind Power Generation (WPG) dataset, evaluating how perturbations in key features (Temperature, Wind Speed (10 m & 100 m), Relative Humidity, and Wind Direction) affect the Root Mean Squared Error (RMSE) and coefficient of determination (R^2^) in temperature and wind power forecasting. Temperature is a major factor in temperature and wind power forecasting. A + 10% increase in temperature results in a 4.1% increase in RMSE (higher prediction error) and a 2.6% decrease in R^2^, meaning the model struggles more. A -10% decrease in temperature improves R^2^ by 2.2%, showing that small temperature fluctuations significantly impact prediction accuracy. Wind Speed (10 m) has a strong effect, but Wind Speed (100 m) has an even greater impact. A + 10% increase in wind speed at 10 m results in a 3.8% increase in RMSE and a 2.3% drop in R^2^. A + 10% increase in wind speed at 100 m has an even more pronounced effect, increasing RMSE by 4.7% and reducing R^2^ by 2.9%. This confirms that higher altitude wind speeds (100 m) have a stronger influence on wind power generation than lower altitude wind speeds (10 m), which is expected because wind turbines operate at higher elevations. Relative Humidity has a moderate effect on forecasting accuracy. A + 10% increase in relative humidity increases RMSE by 2.6% and decreases R^2^ by 1.8%. A -10% decrease in relative humidity improves R^2^ by 1.4%. While humidity affects temperature and atmospheric energy transfer, its effect is not as strong as temperature and wind speed. Wind Direction affects prediction but to a lesser extent than Wind Speed. A + 10% change in wind direction increases RMSE by 3.1% and decreases R^2^ by 1.9%. A -10% change improves R^2^ by 1.6%. Since wind turbines depend on both wind speed and direction, directional changes contribute to power fluctuations, but not as significantly as wind speed itself.

**Table 15:** Sensitivity analysis for temperature and wind power forecasting (WPG dataset).

Feature	Perturbation (%)	RMSE Change (%)	*R*^2^ Change (%)
Temperature	+ 5	+ 2.0	− 1.3
	− 5	− 1.7	+ 1.0
	+ 10	+ 4.1	− 2.6
	− 10	− 3.8	+ 2.2
Wind speed (10 m)	+ 5	+ 1.9	− 1.1
	− 5	− 1.6	+ 0.9
	+ 10	+ 3.8	− 2.3
	− 10	− 3.5	+ 1.8
Wind speed (100 m)	+ 5	+ 2.3	− 1.5
	− 5	− 2.0	+ 1.2
	+ 10	+ 4.7	− 2.9
	− 10	− 4.2	+ 2.5
Relative humidity	+ 5	+ 1.2	− 0.7
	− 5	− 1.0	+ 0.6
	+ 10	+ 2.6	− 1.8
	− 10	− 2.3	+ 1.4
Wind direction	+ 5	+ 1.5	− 0.9
	− 5	− 1.3	+ 0.8
	+ 10	+ 3.1	− 1.9
	− 10	− 2.8	+ 1.6

## Limitation and future work

The accuracy of climate forecasting models heavily depends on the quality and availability of historical climate datasets. Inconsistent, missing, or noisy data can affect the reliability of predictions. The proposed CNN-ResNet50-LSTM model integrates three deep learning architectures, requiring substantial computational resources for training and inference, which may limit its accessibility for researchers with limited hardware. Climate change is influenced by numerous unpredictable factors, making it challenging to model long-term trends accurately. External factors such as natural disasters or anthropogenic activities can cause deviations from historical patterns. The model’s forecasting capability is based on past climate trends, which may not always capture sudden shifts or extreme weather events. The datasets used for training are limited to specific locations and time frames, which may introduce biases that affect the generalization of the model to different climatic regions. While the model performs well on the selected datasets, its effectiveness in entirely different geographic regions with varying climatic conditions remains to be fully tested. Future research will explore the integration of additional environmental factors such as precipitation, humidity, and atmospheric pressure to enhance prediction accuracy. Implementing real-time data streams will allow the model to adapt dynamically to changing climate conditions, improving forecasting accuracy. Extending the study to a more diverse set of global datasets will help assess the model’s generalization and adaptability to different climate zones. Exploring model optimization techniques such as pruning, quantization, or cloud-based solutions can help reduce computational overhead while maintaining high prediction accuracy. Future work may incorporate explainable AI (XAI) techniques to improve model interpretability, ensuring that climate forecasts are not only accurate but also transparent for policymakers and scientists.

## Scalability of the proposed CNN-ResNet50-LSTM model

The proposed CNN-ResNet50-LSTM model is designed to handle large-scale climate datasets efficiently. The combination of CNN, ResNet50, and LSTM ensures that the model can process large climate datasets while capturing both spatial and temporal dependencies. This structure allows it to scale effectively when applied to higher-dimensional climate data. The model demonstrated low training and inference times (Table 6), making it feasible for real-time forecasting applications. Using parallel processing with GPUs, this can be efficiently deployed in operational climate monitoring systems. The model can be trained on additional datasets from different regions without significant modifications and the use of Z-score normalization ensures that the model generalizes well to datasets with different scales. To evaluate the robustness of our model, this study utilized three distinct datasets from different sources, covering varying climatic conditions. The CNN-ResNet50-LSTM model performed consistently well across all datasets, suggesting its ability to generalize to diverse climate scenarios. Since climate conditions vary across regions, retraining the model with location-specific historical data would enhance forecasting accuracy. The model can be fine-tuned with transfer learning to adapt to new environments efficiently. While temperature and wind power are universal climate variables, additional region-specific features can be incorporated to improve forecasting accuracy in specific locations. The model can be integrated with Internet of Things (IoT) networks and weather stations to continuously update forecasts based on real-time sensor data, making it suitable for dynamic climate monitoring.

## Conclusion

Climate change and fluctuations in weather and temperature have emerged as one of the most environmental challenges facing the world today. These changes may comprise the rise in temperature, the increased occurrence of natural catastrophes like storms and floods, as well as the changing patterns of rainfall. All these factors pose a significant threat to daily life, economies, and ecosystems. The proposed model CNN-ResNet50-LSTM integrates CNN, ResNet50, and LSTM deep learning models to provide accurate temperature and wind power forecasts, with the goal of understanding the future behavior of climate change temperature and wind power impact, as well as enhancing the approaches based on deep learning techniques for climate change forecasting. Experimental studies were performed on three different datasets for temperature and wind power: Scada, SA, and WPG. The performance of the proposed model was verified using several metrics, including ${\text{R}}^{2}$, MSE, MAE, MedAE, and RMSE. According to the findings, the proposed deep learning framework performed better than five traditional regression models: DR, ETR, DTR, SGDR, and KRR against the three datasets, with ${\text{R}}^{2}$ scores of 98.84 for wind power forecasting in Scada dataset, 99.01 for temperature forecasting in SA dataset, and 98.58% for temperature forecasting and 98. 53% for wind power forecasting in the WPG dataset.

## Data Availability

Scada dataset: “https://www.kaggle.com/datasets/berkerisen/wind-turbine-scada-dataset”. SA dataset: “https://www.kaggle.com/datasets/esraamadi/saudi-arabia-weather-history”. WPG dataset: “https://www.kaggle.com/datasets/mubashirrahim/wind-power-generation-data-forecasting”.
